# Role of Mushroom Polysaccharides in Modulation of GI Homeostasis and Protection of GI Barrier

**DOI:** 10.1021/acs.jafc.5c00745

**Published:** 2025-03-10

**Authors:** Jianhui Liu, Yi Dai, Wenjian Yang, Zhen-Yu Chen

**Affiliations:** † Collaborative Innovation Center for Modern Grain Circulation and Safety, Jiangsu Province Engineering Research Center of Edible Fungus Preservation and Intensive Processing, College of Food Science and Engineering, Nanjing University of Finance and Economics, Nanjing 210023, China; ‡ School of Life Sciences, The Chinese University of Hong Kong, Shatin, NT, Hong Kong 999077, China

**Keywords:** Edible and medicinal mushroom, Polysaccharides, Gastric barrier, Intestinal barrier, Microbiota

## Abstract

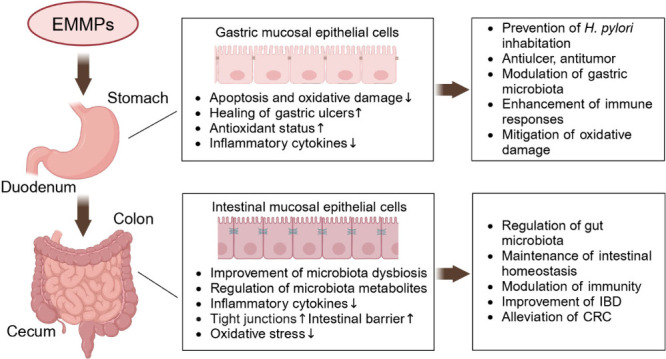

Edible and medicinal mushroom polysaccharides (EMMPs) have been widely studied for their various biological activities. It has been shown that EMMPs could modulate microbiota in the large intestine and improve intestinal health. However, the role of EMMPs in protecting the gastric barrier, regulating gastric microbiota, and improving gastric health cannot be ignored. Hence, this review will elucidate the effect of EMMPs on gastric and intestinal barriers, with emphasis on the interaction of EMMPs with microbiota in maintaining overall gastrointestinal health. Additionally, this review highlights the gastroprotective effects and underlying mechanisms of EMMPs against gastric mucosa injury, gastritis, gastric ulcer, and gastric cancer. Furthermore, the effects of EMMPs on intestinal diseases, including inflammatory bowel disease, colorectal cancer, and intestinal infection, are also summarized. This review will also discuss the future perspective and challenges in the use of EMMPs as a dietary supplement or a nutraceutical in preventing and treating gastrointestinal diseases.

## Introduction

Gastrointestinal (GI) health is essential for overall health. The GI tract serves as an essential site for food intake, digestion, absorption, and elimination of food residue.^1^ Food is processed into a bolus in the oral cavity, then enters the stomach and mixes with gastric acid and digestive enzymes to become chyme. As chyme gradually moves in and through the intestinal tract, it is neutralized by bicarbonate in the duodenum.^2^ Subsequently, the digestion and absorption process primarily occurs in the jejunum and ileum of the small intestine.^3^ The main function of the large intestine is to provide microbiota, absorb water and electrolytes, and expel undigested food residues.^4^ Gut bacteria play a crucial role in metabolizing some unabsorbed food constituents, protecting the mucous layer from pathogens, generating vitamins B and K, and modulating immune function.^5^

The mucosa is a physical and immune defense barrier to protect the GI track against foreign pathogens and toxins in humans.^6^ The gastric mucosa has two primary functions: to lubricate the foods in the stomach and to protect the stomach wall from acid and digestive enzymes. The characteristic feature of the small intestine mucosa is its villous structure, which extends to the lumen, enlarging the surface area of the epithelial cells to facilitate digestion and absorption of nutrients.^7^ Exocrine cells in the mucosa of the small intestine secrete various enzymes, which are essential for the hydrolysis and absorption of fats, carbohydrates, and proteins. Macrophages and dendritic cells underneath the epithelium serve as a major part of the immune system to protect against a variety of extracellular infections and dietary antigens.^8,9^ The mucosa of the large intestine does not have villous projections. The mucosa in the large intestine is the major site of electrolyte and water reabsorption as well as the elimination of feces. Microbiota reside on the mucosa and have a profound impact on health in humans. The stomach and small intestine normally have relatively fewer bacteria than the large intestine. The outer mucous layer of the large intestine is colonized by a large number of microbes. Numerous research results have supported the theory that gut microbiota play a key role in modulating host immunity and maintaining normal metabolism.^10^

Edible mushrooms mainly include *Lentinula edodes*, *Agaricus bisporus*, *Flammulina velutipes*, and *Auricularia auricula*, while medicinal mushrooms refer to those fungi that have certain medicinal value and play a therapeutic or health-care role in human health, such as *Ganoderma lucidum*, *Cordyceps sinensis*, *H. erinaceus*, and *Sanghuangporus sanghuang*. Among them, *G. lucidum*, *C. sinensis*, and *H. erinaceus* have both edible and medicinal value. Edible and medicinal mushrooms are rich sources of bioactive substances, including polysaccharides, alkaloids, steroids, polyphenols, and lipids.^11,12^ Among these, edible and medicinal mushroom polysaccharides (EMMPs) have exhibited great nutritional and healing properties.^13,14^ The most common polysaccharide extracted from both edible and medicinal mushrooms is β-glucan, although the structures, concentration, and complexity may differ. Over the past few decades, extensive research has highlighted the diverse health-promoting activities of EMMPs, including antiobesity, anticancer, antioxidant, anti-inflammation and antidiabetes activity and modulation of lipid metabolism.^15−18^ In recent years, it has been discovered that EMMPs could improve GI health in many ways. On one hand, intestinal microbiota could ferment and utilize EMMPs.^19^ On the other hand, EMMPs could modulate immune system cells such as T and B lymphocytes, macrophages, and dendritic cells (DCs) to initiate immune responses in the host.^20^ Recently, polysaccharides, particularly EMMPs, together with probiotics have been used as a combination to treat various intestinal and colorectal related diseases.^21^ However, their role in protecting the gastric barrier, regulating gastric microbiota, and protecting gastric health cannot be ignored.

This review will explore the unique structural characteristics of EMMPs and discuss their effects on the GI tract with focus on their impact on gastric and intestinal mucosal barriers. It will further illustrate the underlying mechanisms by which EMMPs improve GI health and explore a theoretical basis to develop EMMPs as dietary supplements.

## Structures of EMMPs

Edible and medicinal mushrooms are widely distributed in nature. Due to the complexity of the structure of EMMPs, their specific structures have been sparsely characterized so far. Table 1 lists some species of edible and medicinal mushrooms from which polysaccharides have been extracted. EMMPs have a smaller portion of α-d-glucans present and primarily consist of β-d-glucans with monosaccharide units being linked by glycosidic bonds. The main chains of EMMPs contain β-(1 → 3) linkages with attachment of β-(1 → 6) branches. The majority of the EMMPs discussed in this review have glucose or galactose chains as the backbone and connect mainly through 1 → 6 linkages or 1 → 4 linkages, while some have 1 → 3 linkages. Branches primarily include glucose pyranose or galactose pyranose units, and in some cases, they may include xylose, mannose, or fucose units. Water, alkali, or acid solutions are usually used as the extractants of polysaccharides. The alkali-soluble polysaccharides mainly contain highly branched β-(1 → 3, 1 → 6)-glucan and glycoprotein, while the acid-soluble and alkali-insoluble parts are mainly composed of β-(1 → 6)-glucan, and the yield of polysaccharides is normally higher than that of water extraction.^22^ For example, the polysaccharides isolated from *Pleurotus ostreatus* with 3.8% HCl had a glucose backbone with (1 → 3)-β-Glcp and a small percentage of (1 → 4)-β and (1 → 6)-β linked interior residues,^23^ while the polysaccharides extracted with 1 M NaOH/NaBH_4_ were β-linked glucans showing (1 → 3) and (1 → 6) glycosidic bonds.^24^ Depending on the structural characteristics of polysaccharides such as chemical composition, molecular weight, glycosidic bonds, three-dimensional conformation, and number and structure of branches, the gastrointestinal barrier protection effects may differ. The specific structure–function correlation will be further discussed in the following sections.

**Table 1:**
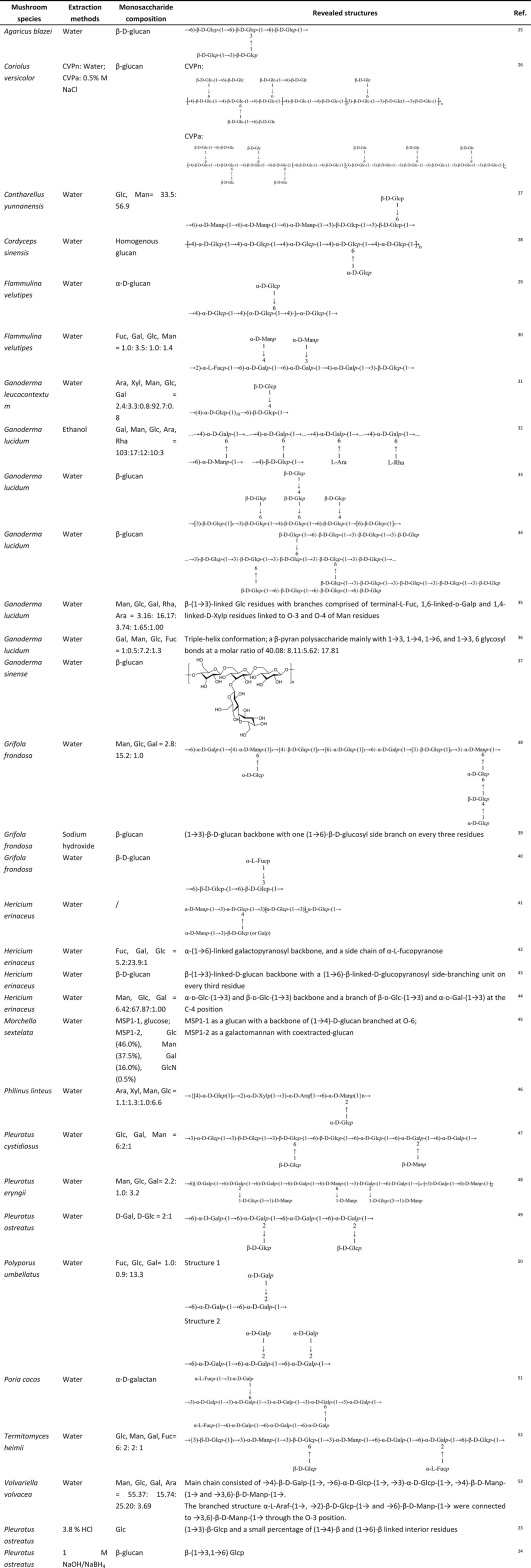
Extraction, Composition, and Structures of EMMPs^a^^23−53^

a Abbreviations: Glc, glucose; Man, mannose; Rha, rhamnose; Gal, galactose; GalA, galactonic acid; GlcA, glucuronic acid; Xyl, xylose; Fuc, fucose; Ara, arabinose; GlcN, glucosamine.

## Digestion, Absorption, and Utilization Pathways of EMMPs

Polysaccharides can be divided into two categories, namely, digestible and indigestible polysaccharides. The digestible polysaccharides such as starch can be hydrolyzed to release simple sugars (glucose) by relevant enzymes in the GI track, whereas indigestible polysaccharides can reach the large colon where they are broken down by gut microbiota to small molecular compounds such as short chain fatty acids (SCFAs).^54^ β-Glucan is one of the major indigestible polysaccharides in EMMPs. When ingested, EMMPs are not broken down by the gastric acidic environment and digestive enzymes in the gastrointestinal tract.^55^ Specifically, in simulated digestive systems, including both gastric and intestinal regions, most acquired EMMPs showed no significant changes in the main functional groups and molecular weight. The production of free monosaccharides was also not observed. Ma et al. studied the digestion process of *Pleurotus eryngii* polysaccharides by *in vitro* digestion models and found that the molecular weight and overall structure remained unchanged, suggesting that the polysaccharide remained intact and was not degraded during the digestion process.^56^ A metabolomic study was performed on the systemic metabolism of *Grifola frondosa* and *Inonotus obliquus* polysaccharides in mice by analyzing the metabolic profiles in serum, colon, intestinal contents, and feces, finding that these polysaccharides significantly affected some pathways including amino acid metabolism, the tricarboxylic acid cycle, and purine and pyrimidine metabolism. Specifically, *G. frondosa* polysaccharides exhibited more pronounced regulation on carbohydrate metabolism, while *I. obliquus* polysaccharides had a more significant impact on tryptophan metabolism.^57^*L. edodes* polysaccharides, primarily composed of β-glucans, passed through the intestine relatively intact and formed a gel upon reaching the mucosal surface, subsequently modulating bile salt absorption and altering the gut microbiota.^58^ These findings indicated that EMMPs could reach the large intestine intact where they were transformed by the intestinal microbiota into a range of easily absorbable small molecules, primarily SCFAs and gases such as hydrogen, carbon dioxide, and methane).^59^ These metabolites were further utilized and eventually entered the bloodstream.^60^ The interaction between EMMPs and the bacteria in the colon suggested their potential role in regulating gastrointestinal health. However, the specific structural changes of EMMPs when they were passing through the gastrointestinal tract and how they play beneficial roles are not yet fully understood.

In addition to regulating health, EMMPs have also served as substrates for nanomaterials.^61^ The total concentration of certain drugs in the bloodstream after digestion and absorption, known as the actual oral bioavailability, ultimately determines their therapeutic activity. Therefore, achieving higher oral bioavailability was the most crucial factor in realizing the intended therapeutic effects of drugs. Nanoparticles based on EMMPs possess excellent properties such as nonimmunogenicity, biodegradability, and biocompatibility, making them ideal nanocarriers for drug delivery. By altering their physicochemical properties, EMMP-based nanoparticles enhanced drug uptake rates.^62^

## Pathogenesis of Gastrointestinal-Related Diseases

The GI mucosal barrier provides various defenses for the body, including physical, chemical, biological, and immune protections. The mucosa comprises physical components (the epithelial cell layer), chemical elements (the mucus layer), immune components (various immune cells mainly located in the subepithelial layer), and biological barriers (gastrointestinal microbiota).^63^ These four components are closely interconnected, forming a collective entity that directly or indirectly safeguards the gastrointestinal tract.

### Gastric Barrier Damage

#### Composition of the Gastric Barrier

**Figure 1:**
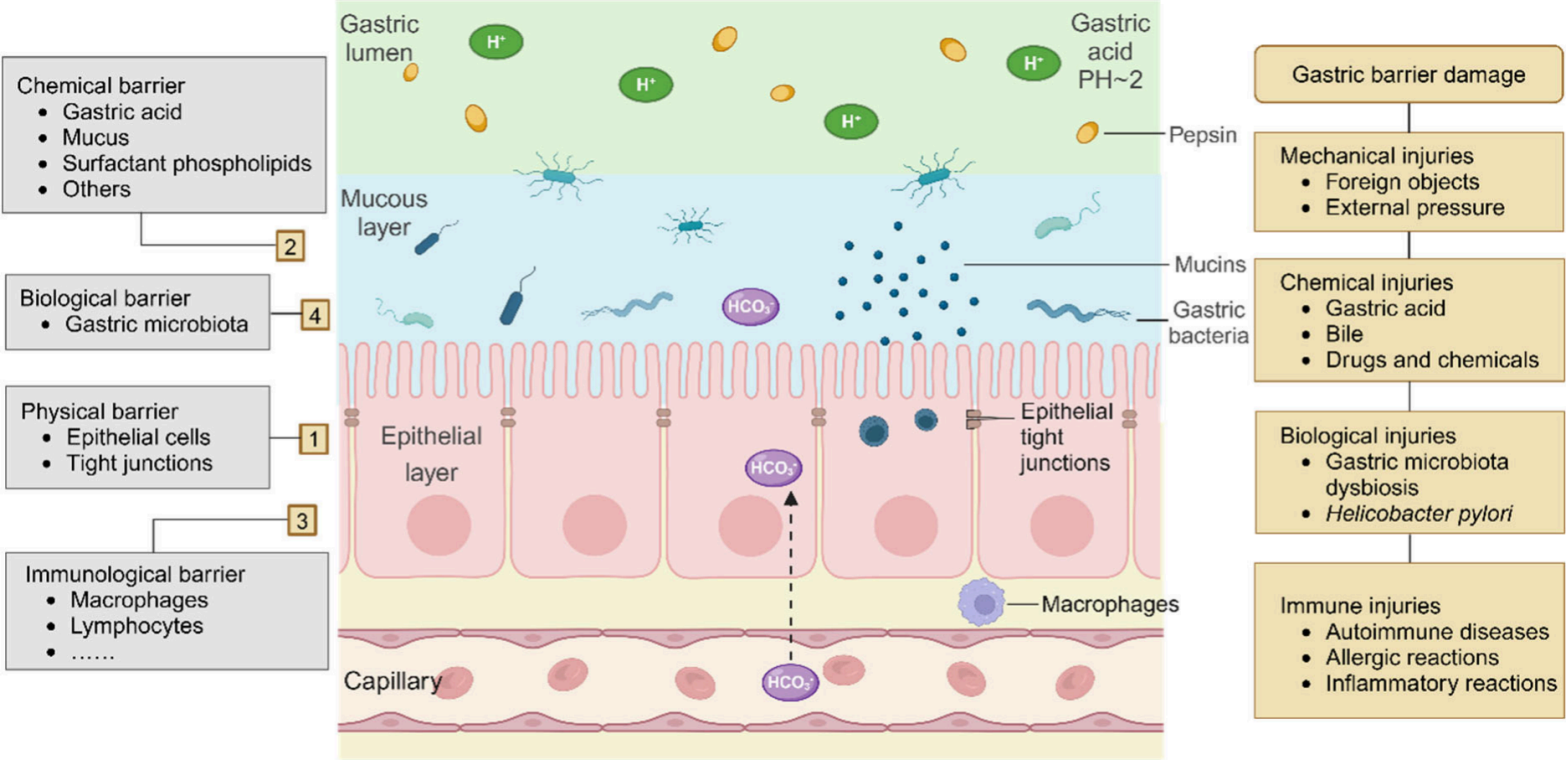
Structure and composition of gastric barrier and factors contributing to its damage.

The gastric mucosa has four levels of protective barriers (Figure 1). The first level refers to the epithelial cells and intercellular spaces of the gastric mucosa. They form a cell layer through tight connections, preventing harmful substances from entering the mucosal tissue. These cells also participate in the secretion of mucus and other protective substances, also known as the physical barrier.^64^ The second level refers to various defensive substances distributed on the surface of the gastric mucosal epithelium, including gastric acid, mucus, surfactant phospholipids, and other antimicrobial substances. These substances are secreted by the gastric mucosa, known as the chemical barrier.^65^ The third level is the mucosal immune barrier, mainly including macrophages and lymphocytes, which participate in immune reactions and identify and eliminate invading harmful microorganisms.^66^ The fourth level involves the gastric microbiota, which constitute the biological barrier. Due to the high acidity environment in the stomach that is unfavorable for the growth of most microorganisms, specific gastric microbial groups (mainly composed of Firmicutes, Bacteroidetes, actinomycetes, *Clostridium*, and Proteobacteria) better adapt and survive in this environment, competitively preventing the invasion and proliferation of other harmful microorganisms.^67^ The gastric microbiota also interact with the immune system, regulating the immune balance and thereby influencing the overall immune response.^68^ However, the important role played by the gastric mucosal barrier, especially gastric microbiota, in regulating human health is often overlooked.

The integrity of the gastric barrier is a complex system influenced by various interacting endogenous and exogenous factors. The main endogenous factors are as follows.^69,70^ The first is gastric mucosal physiological conditions, such as the structure and function of the mucous layer and epithelial cells and the chemical composition of the mucus, which directly impact the integrity of the barrier. The second endogenous factor is gastric acidity because it plays a vital role in maintaining the acidic environment in the stomach. Moderate gastric acidity helps eliminate harmful microorganisms that are ingested and is also an essential component of gastric mucus. The third endogenous factor is the immune system. The presence of immune cells in the stomach allows for the recognition of and response to potential pathogenic microorganisms. The health of the immune system is vital to the protective function of the gastric barrier. The fourth of the endogenous factors is the gastric microbiota. These microorganisms contribute to maintaining the acidic environment in the stomach, providing protection against invasion and proliferation of harmful microorganisms. Additionally, they participate in regulating gastric fluids, promoting food breakdown, and digestion.

The integrity of the gastric barrier is also influenced by four exogenous factors.^71−74^ The first factor is diet and nutrition: Components in the diet, such as antioxidants and dietary fiber, can influence the health of the gastric environment and mucosa. Poor dietary habits may lead to damage to the mucosal barrier. The second exogenous factor is the use of drugs and medicines. Some drugs, especially nonsteroidal anti-inflammatory drugs (NSAIDs), antibiotics, etc., may exhibit some adverse impacts on the gastric barrier, resulting in gastric mucosal injury or ulcer formation. The third exogenous factor is infection and inflammation. Infections or inflammatory conditions in the stomach directly influence the gastric barrier, especially infections caused by *Helicobacter pylori* may lead to inflammation and ulcer formation. The fourth exogenous factor is the environment. Exposure to harmful environments, such as smoking and alcohol consumption, may also have negative effects on the gastric barrier.

#### Mechanisms of Gastric Barrier Damage

The damage to the gastric barrier involves the actions of various attacking factors and can be summarized and categorized from four perspectives: physical, chemical, biological, and immune barrier injuries (Figure 1). Mechanical injuries fall under the physical category and are primarily caused by friction or compression of certain foreign objects in the stomach (such as large food particles or medications) or external pressure, leading to mechanical damage to local tissues, such as stretching, tearing, or deformation of the mucosa.^75^ Gastric acid, bile, and drugs and chemicals are the main factors that cause the chemical injuries. First, the secretion of gastric acid is primarily stimulated by histamine, gastrin, and acetylcholine, while it is inhibited by somatostatin. Excessive secretion of gastric acid or imbalance of mucosal defense can cause the direct contact of gastric acid with the stomach wall and result in gastric mucosa damage. Under strong acidic conditions with a pH < 3, gastric acid not only activates pepsinogen into pepsin, initiating self-digestion, but also causes hydrogen ions to infiltrate, directly damaging the gastric mucosa.^76^ Second, bile refluxes into the stomach and washes away the mucus in the stomach, weakening the isolation effect of the mucus. The chemical components such as bile salts in bile may dissolve the lipid layer and cause esterification of the epithelial cell membrane, disrupting the mucous and gastric mucosal barriers, resulting in gastric mucosal inflammation and ulcers.^77^ Third, prolonged or high-concentration use of certain drugs (such as NSAIDs) and chemical substances like alcohol (acidic or alkaline) may cause tissue damage through direct toxicity or by inducing inflammatory reactions. NSAIDs can lead to gastric mucosal damage by depleting prostaglandins derived from cyclooxygenase.^78^ Alcohol can dissolve the gastric mucus layer, increase mucosal permeability, and induce oxidative stress.^79^

Biological injuries mainly involve changes in the gastric microbiota, including infections by *H. pylori* and other bacteria, viruses, and fungi. These infections can produce toxins and enzymatic substances with toxicity, leading to gastritis, ulcers, and chronic gastric disorders.^80^ Besides, *H. pylori* infection can cause changes of the structure of the gastric microbiota, and eradication of *H. pylori* can reverse gastric microbiota dysbiosis and enrich the dominant commensal gastric bacteria.^81^ This suggests that dysbiosis of the gastric microbiota can damage the gastric microbial barrier and probably causes the occurrence of gastric diseases.

Immune injuries include autoimmune diseases, allergic reactions, and inflammatory reactions. First, diseases like autoimmune gastritis involve the immune system attacking gastric mucosal tissues, leading to tissue destruction and functional abnormalities.^82^ Second, allergic reactions of the immune system caused by certain foods or substances may cause inflammation and damage. Third, when the body is invaded by pathogens, sentinel cells (such as macrophages or other inflammation-related cells) release inflammatory mediators, increase granulocyte infiltration, generate oxygen free radicals, and cause inflammatory damage to the gastric mucosa.^83^

Gastric barrier damage is often accompanied by an inflammatory response and an increase in free radicals. The mechanisms causing gastric mucosal lesions vary with different attacking factors, but their fundamental basis lies in the imbalance between defensive and aggressive factors. When aggressive factors are intensified or defensive factors weaken, the gastric barrier is compromised, leading to the permeation of hydrogen ions, release of histamine from mast cells, and stimulation of parietal cells to accelerate gastric acid secretion.^84^ This series of reactions will cause microcirculatory disturbances in the gastric mucosa, simultaneously leading to inflammatory reactions such as congestion and edema in the short term and formation of erosion and ulcers in the long term.^85^ When the stomach undergoes various stress reactions, a large amount of oxygen free radicals are generated, resulting in circulatory disturbances within the body. Simultaneously, the free radicals combine with polyunsaturated fatty acids in the membrane, causing lipid peroxidation damage and forming lipid peroxidation products such as malondialdehyde (MDA).^86^ Inflammation-induced damage to the superficial layers of the gastric mucosa may result in superficial gastritis and erosive gastritis, while damage to the deeper layers may lead to gastric ulcers and atrophic gastritis. Atrophic gastritis may gradually develop from superficial gastritis, accompanied by intestinal metaplasia and atypical hyperplasia, among other precancerous lesions, eventually evolving into gastric cancer.

### Intestinal Barrier Damage

#### Composition of the Intestinal Barrier

**Figure 2:**
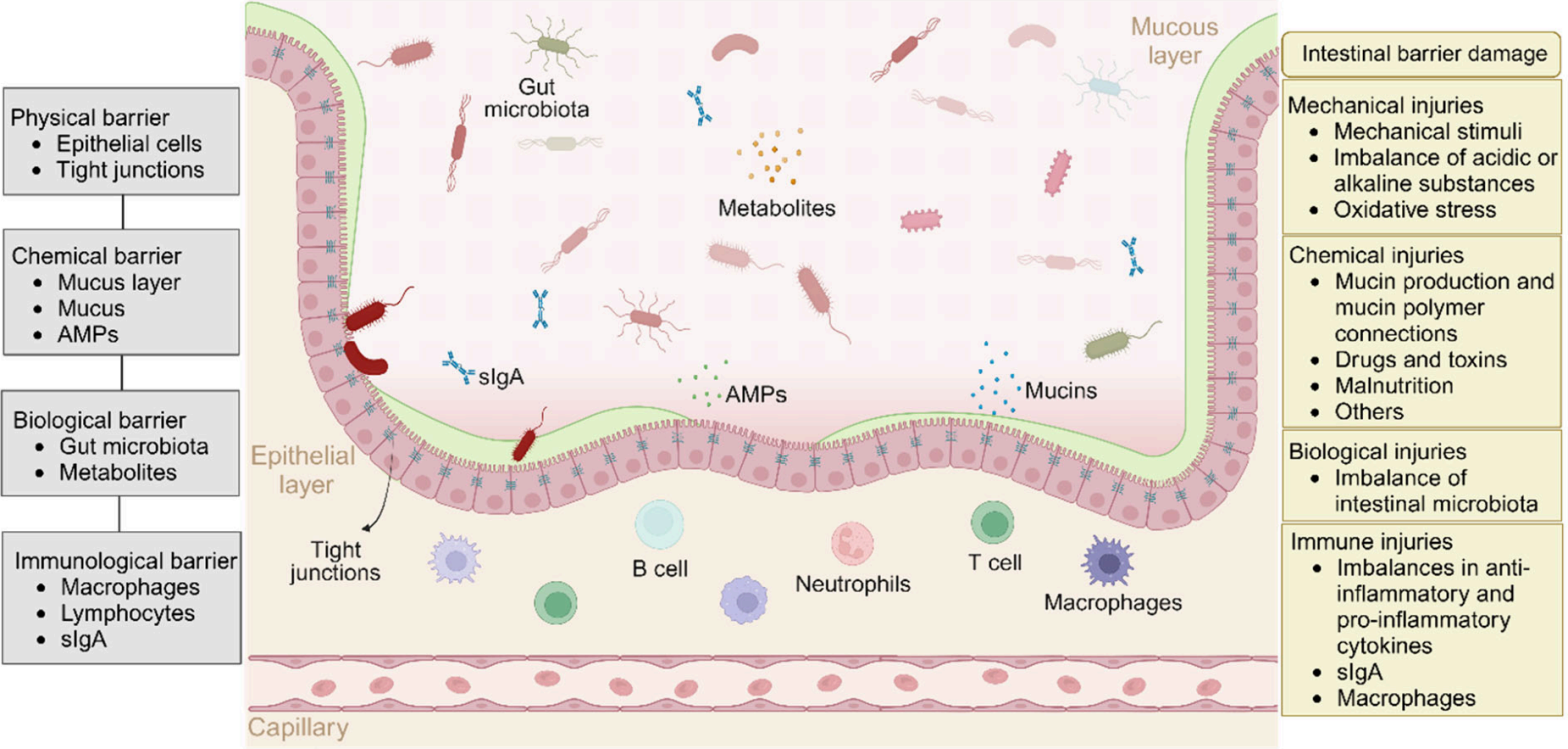
Composition and factors contributing to damage of intestinal barriers. Abbreviations: slgA, secretory immunoglobulin A; AMPs, antimicrobial peptides.

The intestinal mucosa also has four levels of protective barriers, namely, physical, chemical, immune, and biological barriers (Figure 2).^87^ The essential component of the intestinal physical barrier refers to the morphology of the mucosa, directly influencing the absorption of nutrients. An increase in villus height implies numerous mature villous cells, resulting in a greater absorptive surface area and enhanced absorption capacity.^88^ Intestinal crypts primarily produce cells and sustainably replenish epithelial cells on the villi. An increase in crypt depth suggests a slowing down of the maturation speed and a reduction in the secretion capacity of villous epithelial cells. The ratio of villus height to crypt depth is a vital indicator to assess the morphology, integrity, and function of the intestinal mucosa.^89^ The intestinal epithelial cells (IECs) are connected by tight intercellular junctions, forming a continuous layer.^90^ Intercellular connections consist of tight junctions (TJs), adherent junctions (AJs), and desmosomes.^91^ TJs are one of the most important barrier systems that present on the surface of IECs, mainly including four transmembrane proteins: zonula occludens (ZOs), occludins, claudins, and Jams.^92^ Besides, TJs are linked to the cytoskeleton supporting the IECs, and they can establish a dynamic barrier system that directly controls intestinal permeability.^93^

The intestinal chemical barrier includes the mucus layer, mucins, and antimicrobial peptides (AMPs), as well as other substances secreted by IECs.^94^ The mucus layer is produced by mucosal cells and is mainly composed of mucins, i.e., mucin 2 (Muc-2), which have the function of adhering to microbes and preventing them from attaching to the mucosal surface.^95^ Through the production and secretion of mucus, a thick layer forms, thereby blocking the invasion of microbes and protecting the mucosa from the effects of irritating substances. The intestinal mucosa secretes antimicrobial substances, such as AMPs, which have a direct killing or inhibitory effect on microbial growth.^96^ In the intestinal tract, prostaglandins are vital for protecting the mucosa from damage. They are lipid substances produced by mucosal cells and function in regulating mucosal blood flow, promoting mucus secretion, and maintaining the structural integrity of the mucosa.^97^

The immune barrier of the intestinal tract is mainly composed of gut-associated lymphoid tissue (GALT), immune cells, and immunoglobulins. GALT is primarily located beneath the intestinal mucosal epithelium, including Peyer’s patches and intestinal intraepithelial lymphocytes (IELs).^98^ By monitoring and responding to potential pathogens, it maintains the immune balance within the intestinal tract. The generation and activation of immune cells, mainly including macrophages, T cells, B cells, and neutrophils, in the intestinal tract primarily occur in the GALT.^99^ Through activation of the immune system, the immune cells identify and clear invading pathogens, regulate inflammatory responses, and maintain a stable microbial balance in the intestinal tract. Secretory immunoglobulin A (sIgA) is primarily secreted by lymphocytes and plasma cells, which are distributed on the surface of the mucosa. It is the most abundant immunoglobulin in the intestinal secretions and is the main factor preventing the invasion of pathogens and regulating immune responses in the intestinal tract.^100^

The combination of symbiotic microorganisms with the mucosa forms the biological barrier of the intestine. Normal flora colonize the intestinal tract, affording necessary nutrients, avoiding the colonization and expansion of pathogenic bacteria, and maintaining the microbial balance within the intestinal tract. Simultaneously, they boost mucosal immunity, facilitate the growth and maturation of immune organs, and regulate immune balance.^101^ The most abundant phylum in the intestine is Firmicutes, accounting for approximately 65%. The second most prevalent is Bacteroidetes, constituting around 25%. There are also smaller quantities of Actinobacteria, Proteobacteria, Clostridia, and Verrucomicrobia.^102^ Molecules secreted by probiotics (such as *Lactobacillus* and *Bifidobacterium*), including surface layer proteins, capsule polysaccharides, flagella, and pili, interact with pattern recognition receptors (PRRs) in the immune system of the host. This interaction triggers the activation/inhibition of various signaling pathways, inducing the production of cytokines or inhibiting cell apoptosis, subsequently reducing inflammatory response and promoting intestinal epithelial function.^103^ Metabolites produced by probiotics, including secreted proteins, indoles, organic acids, extracellular vesicles, SCFAs, and bacteriocins, also protect the functional integrity of the epithelial barrier through interacting with various receptors.^104^

#### Mechanisms of Intestinal Barrier Damage

Various factors such as unhealthy diet, diseases, and stress can impact the intestinal barrier, leading to mucosal atrophy, increase of intestinal permeability, epithelial cell injury, impairment of the intestinal immune system, and dysbiosis of the gut microbiota. The damage to the intestinal barrier is often manifested as a comprehensive injury to various physical, chemical, biological, and immune barriers (Figure 2).

The main components of the physical barrier are IECs and TJs between the cells. Damage to the physical barrier of the intestine can be caused by intense intestinal movements, trauma, surgery, or other mechanical stimuli, resulting in mucosal tearing, ulcers, or bleeding.^105^ Imbalances of acidic or alkaline substances may alter the pH of the intestinal environment, resulting in cell membrane damage and structural changes in cells, increasing barrier permeability and affecting intestinal mucosal health. Additionally, the excessive accumulation of reactive oxygen species (ROS) induces oxidative stress, which disrupts TJ complexes between cells via different signaling pathways. Mitogen-activated protein kinase (MAPK), c-Jun N-terminal kinase (JNK), extracellular signal-regulated kinase (ERK), and protein kinase C (PKC) are the most common signaling molecules. Oxidative stress also disrupts AJs and protein scaffolds by redistributing TJ proteins, interrupting protein modifications, and impairing intestinal epithelial barrier function.^106^

Different types of mucins in the intestine and the mucus layer covering IECs can act as a chemical barrier to protect IECs from invasion by pathogens and foreign harmful substances.^107^ Damage to the intestinal mucous layer caused by decreased mucin secretion and impaired mucin polymer connections will lead to a variety of intestinal diseases, such as inflammatory bowel disease (IBD), ulcerative colitis (UC), and type 2 diabetes mellitus (T2DM).^108−110^ The long-term or excessive use of certain drugs and toxins, such as NSAIDs, alcohol, and chemicals, may directly damage the intestinal mucosal cells. For example, cyclooxygenase inhibitors like indomethacin inhibit the secretion of prostaglandins in the intestinal mucosa, increase mucosal epithelial permeability, and cause clinical intestinal lesions.^111^ Chemotherapy and anti-transplant rejection drugs also damage the mucosal barrier.^112^ Worse, malnutrition not only reduces transcription levels, protein synthesis, and cell proliferation of intestinal epithelial cells but also results in the reduction of the mucosal layer thickness, mucosal atrophy, and a decrease in mucosal enzymatic activity.^113^ Simultaneously, the decrease in the chemical bactericidal ability of gastric acid, bile, mucopolysaccharides, lysozymes, glycoproteins, and intestinal fluids facilitate the proliferation of intestinal pathogens, resulting in excessive bacterial growth in the intestine.^114^

The damage to the biological barrier of the intestine primarily refers to disruption of the intestinal microbiota. Various microorganisms residing in the intestinal tract play a crucial role in digestion, providing energy, regulation of immune responses, and prevention of invasion by harmful pathogens.^115^ A healthy intestinal microbiota environment mainly includes Firmicutes, Bacteroidetes, and small amounts of Actinobacteria and Proteobacteria.^116^ Various factors such as diet, stress, disease, drug abuse, and poor lifestyle may cause imbalances in intestinal microbiota. In clinical settings, the prolonged use of broad-spectrum antibiotics usually results in dysbiosis of the microbial community in the intestine, subsequently causing disorders of the intestinal mucosal biological barrier.^117^ Moreover, the misuse of antibiotics can enhance the growth of drug-resistant bacterial populations in the intestine, allowing the invasion of exogenous microbial communities. This compresses the growth space for the original microbial community, causing an imbalance in the intestinal microbial structure and ultimately damaging the intestinal mucosal barrier.^118^ Additionally, long-term smoking markedly changes the composition and activity of the microbial community in the colon.^119^ Compared to nonsmokers, smokers tended to have lower relative abundances of Actinobacteria and *Lactobacillus*.^120^ Infections and food poisoning caused by pathogenic bacteria, such as *Salmonella*, *Shigella*, and *Escherichia coli*, release a large number of enterotoxins, disrupting the balance of the microbial community and the intestinal permeability.^121^

Intestinal immune barrier damage typically affects the integrity and barrier function of TJs. IECs facilitate immune responses and increase their integrity via modulating the homeostasis of mucosal immune cells and interacting with dietary and microbial antigens.^122^ Imbalances in anti-inflammatory and proinflammatory cytokines can destroy the barrier integrity.^123^ Signaling pathways induced by toll-like receptor 4 (TLR4), interleukin-1 beta (IL-1β), interleukin-13 (IL-13), tumor necrosis factor alpha (TNF-α), and interferon gamma (IFN-γ) compromise barrier function, while interleukin-10 (IL-10) and transforming growth factor beta (TGF-β) serve as anti-inflammatory factors to protect the intestinal barrier.^124^ When traumatic infection or shock occurs, numerous inflammation-activating factors, such as endotoxins (produced by Gram-negative bacteria) and proinflammatory factors, activate various intracellular signaling pathways,^125^ particularly NOD-like receptor family pyrin domain containing 3 (NLRP3), nuclear factor kappa B (NF-κB), signal transducer and activator of transcription 3 (STAT3), ERK, MAPK, activator protein 1 (AP-1), etc. This activation occurs through combining specific receptors and secreting mutually promoting inflammatory mediators, activating polymorphonuclear leukocytes, and polarizing macrophages. This strong stimulation affects vascular endothelial cells, releasing a large amount of oxygen-free radicals, creating a vicious cycle that further results in intestinal mucosa damage. The involved inflammatory mediators such as cytokines (TNF-α, IL-1β, etc.), chemokines include interleukin-8 (IL-8) and monocyte chemoattractant protein-1 (MCP-1), leukotrienes, platelet-activating factor (PAF), and increased prostaglandins.^126^ Besides, trauma and burns result in a reduction in the number and quality of plasma cells in the lamina propria of the intestinal mucosa, leading to reduced secretion of sIgA. This reduction makes it relatively easier for bacteria and toxins to invade, subsequently weakening the function of the immune barrier of the intestine. The function of macrophages is also one of the factors that affects the intestinal barrier. Under normal conditions, macrophages have the function of phagocytizing bacteria and endotoxins and presenting antigens. However, once traumatized, macrophages will become dysfunctional and lose their bactericidal function. This impairment in macrophage function contributes to a compromised ability to eliminate pathogens and antigens, further contributing to the overall weakening of the intestinal immunity in the aftermath of trauma and shock.^127^

## Gastroprotective Effects and Possible Mechanisms of EMMPs

Gastric mucosal barrier damage is a common pathogenesis of gastric diseases. It not only involves mucosal damage such as erosion and ulcers but also causes abnormalities in gastrointestinal motility, digestion and absorption, and intestinal microecology. Factors such as stress, smoking, alcohol, nutritional deficiencies, infections, and frequent use of nonsteroidal anti-inflammatory drugs can stimulate gastritis and pathological changes in the gastric mucosa and further develop into gastric ulcers. As chronic inflammation of the gastric mucosa persists for a long time and the mucosa is damaged and repaired repeatedly, precancerous lesions such as intestinal metaplasia are prone to occur. Without intervention, it will gradually evolve into gastric cancer. In recent years, numerous studies have confirmed that EMMPs have the function of regulating gastric health, mainly in the following four aspects: (1) protection of the gastric mucosal barrier; (2) antigastritis and anti-inflammation activity; (3) anti-gastric-ulcer activity; (4) anticancer activity. Table 2 summarizes the various mechanisms by which EMMPs are gastroprotective.

**Table 2:** Gastroprotective Activities and Associated Mechanism of EMMPs^a^

Polysaccharides	Source	Subject	Treatment	Mechanism and effect	ref
Gastric Mucosa Injury					
EP-1, mainly composed of (1→3)-linked Glup units with approximately 10% each of (1→)-Manp units and (1→3,4)-Glup units and 1.5% of (1→3,4)-Galp units	*H. erinaceus*	H_2_O_2_ treated human gastric mucosa epithelium cells GES-1	0.03, 0.1, 0.3 mM	↑oxygen radical absorbance capacity (ORAC); DPPH, superoxide and hydroxyl radicals; ↓apoptotic cell death by inhibiting activation of apoptotic cellular signals within mitochondria-dependent apoptotic pathways	(128)
HEP_W1_, composed of fucose (6.13%), glucose (72.28%) and galactose (21.59%), Mw: 5.163 kDa	*H. erinaceus*	H_2_O_2_ treated human gastric mucosa epithelium cells GES-1	125, 250, 500, 1000, 2000, 4000 μg/mL	↑antioxidant capacity; Regulated intracellular antioxidant enzyme system; ↓apoptosis, cell cycle, ROS, DNA damage	(129)
HEP_N_, composed of mannose (5.13%), glucose (43.02%), and galactose (51.85%), Mw: 12.713 kDa	*H. erinaceus*	H_2_O_2_ treated human gastric mucosa epithelium cells GES-1	125, 250, 500, 1000, 2000 μg/mL	↓oxidative damage; ↑cell proliferation; ↓cell necrosis, ROS levels; Regulated mitochondrial membrane potential and maintained mitochondrial membrane permeability	(130)
Mycelium polysaccharide (HMP) and fruiting body polysaccharide (HFP) both have the pyran ring structure and the same main functional groups. The morphology of HFP is more stable.	*H. erinaceus*	alcohol-induced gastric mucosal injury, GES-1 cells and rats	19.8 mg/kg bw	Pro-migration effect and proliferation on GES-1 cell; ↓ethanol-induced acute gastric mucosal injury; HMP showed better activity than HFP	(131)
β-glucan H6PC20 ((1→3), (1→6)-β-d-glucan, Mw: 2390 kDa); α-heteropolysaccharide HPB-3, Mw: 15 kDa	*H. erinaceus*	ethanol-induced gastric mucosa injury, rats	125, 250, 500 mg/kg bw	↑defense and repair factors (EGF, bFGF and PGE2); ↓inflammatory cytokines (IL-1β and TNF-α) and MDA; ↓oxidative injury	(132)
POPw, contained 97.1% total sugar, composed of glucose (52.3%), galactose (25.8%), mannose (10.0%), rhamnose (6.1%), and arabinose (5.2%), Mw: 23 kDa	*P. ostreatus*	acetic acid-induced gastric lesions, rats	100, 200, and 400 mg/kg bw	↑mucus synthesis and prostaglandin production; ↑level of GSH and activity of SOD; ↓content of TBARS	(133)
FVP and U-FVPs	*F. velutipes*	Ethanol-induced injury in GES-1 cells	10, 20, 40, 80 μg/mL	↓ROS production; ↑mRNA expression of TFF2, PGE2, EGF, and TGF-β1	(134)
Gastritis					
EP-1, composed of glucose, mannose, and galactose, with a backbone of α-d-glucan(1→3) and β-d-glucan(1→3), MW: 3100 Da	*Hericium*	GES-1 cells transformed by MNNG	100 or 500 μg/mL	↓growth of MC cells; Interfered with the MC cells by inducing cell cycle arrest	(41)
HEP, Mw: 197 and 20 kDa	*H. erinaceus*	*H. pylori*	20 μg/mL	↓*H. pylori*	(135)
Gastric Ulcer					
GLPs isolated from the fruiting bodies of *G. lucidum*	*G. lucidum*	indomethacin-induced lesions, rats	250 and 500 mg/kg bw	↓TNF-α gene expression; ↑ODC activity	(136)
TEps, with total glucan content 17.81 g/100 and 0.79 g α-glucan and 17.02 g β-glucan	*T. eurrhizus*	indomethacin induced gastric ulceration, mice	1, 10, 20, and 40 mg/kg bw	↓MPO activity; protected mucosal mucin; ↑synthesis of PGE2 by modulation of COX-1 and COX-2 expression; Shifted cytokine expression from pro- (TNF-α, IL-1β) to anti-inflammatory (IL-10)	(137)
Concentrate polysaccharides (EP) and EtOH-soluble fraction (ES)	*H. erinaceus*	ethanol-induced ulcer mice	1.2 and 2.5 g/kg bw	↓ulcerated area	(138)
RP-S, a (1→6)-β-d-glucan, whose backbone was composed of →6)-β-d-Glcp-(1→ residue and branched with T-β-d-Glcp-(1→ residue at O-3 position	*H. erinaceus*	Acetic acid-induced gastric ulcer, rats	100, 200, and 400 mg/kg·bw	↓levels of IL-6, TNF-α, and malondialdehyde and myeloperoxidase activity; ↑releases of NO, PGE2, EGF, VEGF and bFGF and SOD activity	(139)
Total polysaccharides 7.2 mg/mL	*A. rugosum*	ethanol-induced, indomethacin-induced gastric ulcer and pyloric ligation model, rats	50, 100, and 200 mg/kg bw	↓ulcerous area; ↓TNF-α, IL-6, IL-1β; ↓Serum NO; ↑PGE2 concentration; ↓Gene expression of inflammasome NLRP3, and nuclear translocation of NF-κB P65	(140)
HECP and HERP	*H. erinaceus*	Ethanol-induced gastric mucosal lesion and pylorus ligation-induced gastric ulcer, rats	100, 200, and 400 mg/kg bw	↓ethanol-induced gastric mucosal lesion and pylorus ligation-induced gastric ulcer; ↓Levels of TNF-α, IL-1β, and MPO activity; ↑antioxidant status of gastric tissue; ↑Defensive factors (NO, PGE2, EGF)	(141)
GLPs with different molecular weights, 100 kDa, 10 kDa, and 1 kDa	*G. lucidum*	ethanol-induced acute gastric injury, rats	100, 200, and 400 mg/kg bw	Improved symptoms of gastric mucosal congestion and bleeding; ↓serum myeloperoxidase, inflammatory factor, and histamine; ↑antioxidant activity and defense factors (NO and EGF)	(142)
PS, purity 98.8%, an average molecular size of 4.85 × 10^5^ Da, consisted of glucose (61.2%), xylose (15.5%), fructose (14.4%), galactose (4.8%), and rhamnose (4.1%) linked together by β-glycosidic linkages	*G. lucidum*	Acetic acid-induced ulcers, rats	0.1, 0.5, or 1.0 g/kg	↑ulcer healing; ↑gastric mucus and prostaglandin levels	(143)
Gastric Cancer					
POMP2, Mw: 29 kDa	*P. ostreatus*	BGC-823 xenograft tumor model, mice	50, 100, and 200 mg/kg	↓the colony forming capacity and the invasive capabilities of BGC-823 cells; ↓tumor weight and volume	(144)
EP-1, composed of glucose, mannose and galactose, with a backbone of α-d-Glc(1→3) and β-d-Glc(1→3), 3100 Da	*Hericium*	GES-1 and MC cell line transformed by MNNG	0.0625, 0.12, 0.25, 0.5, 1, and 3 mg/mL	Induced cell apoptosis and cell cycle arrest at the G0/G1 phase; Modulated the expression of Bax, Bcl-2, and caspase-3	(145)

a Abbreviations: DPPH, 2,2-diphenyl-1-picrylhydrazyl; ROS, reactive oxygen species; EGF, epidermal growth factor; bFGF, basic fibroblast growth factor; PGE2, prostaglandin E2; IL-1β/6/10, interleukin-1 beta/6/10; TNF-α, tumor necrosis factor-alpha; MDA, malondialdehyde; GSH, l-glutathione; SOD, superoxide dismutase; TBARS, thiobarbituric acid reactive substance; MNNG, *N*-methyl-*N*′-nitro-*N*-nitrosoguanidine; *H. pylori*, *Helicobacter pylori*; ODC, ornithine decarboxylase; MPO, myeloperoxidase; COX-1/2, cyclooxygenase-1/2; NO, nitric oxide; VEGF, vascular endothelial growth factor; NLRP3, NOD-like receptor family pyrin domain containing 3; NF-κB, nuclear factor-kappa B; Bax, BCL-2-associated X protein; Bcl-2, B-cell lymphoma-2; Caspase-3, cysteine-containing aspartate-specific protease-3; TFF2, triglyceride factor 2; TGF-β1, transforming growth factor β1; FVP, *F. velutipes* polysaccharides; U-FVPs, FVP ultrasonic modification products; HECP, *H. erinaceus* crude polysaccharide; HERP, *H. erinaceus* refined polysaccharide.

### Effect of EMMPs on Gastric Mucosal Injury

Gastric mucosal injury will affect gastric function and self-repair ability of the gastric mucosa and may cause gastritis, gastric ulcer, gastric cancer, and other related diseases. Various studies have indicated that EMMPs have a protective effect on the gastric mucosa (Table 2). Generally, EMMPs can provide a protective effect for the gastric mucosal barrier by improving physical and chemical barriers, enhancing antioxidant effects, and enhancing immune barriers.

When various stress reactions, chemical influences, or gastric mucosal ischemia occur in the stomach, a large amount of oxygen free radicals are generated and react with polyunsaturated fatty acids within the membrane, resulting in the production of lipid peroxidation products such as acetaldehyde.^146^ In human gastric mucosal epithelial GES-1 cells, *H. erinaceus* polysaccharides could prevent apoptosis and oxidative damage induced by hydrogen peroxide (H_2_O_2_), thus demonstrating good gastric mucosal protection effects.^128−130^ Besides, *H. erinaceus* polysaccharides can effectively alleviate ethanol-induced acute damage, mainly by promoting the defense function of gastric mucosa, reducing inflammatory response, mitigating oxidative damage, and promoting cell migration to protect the gastric mucosa.^131,132^*P. ostreatus* polysaccharides significantly inhibited acetic acid-induced gastric injury by increasing gastric mucosal blood flow and enhancing mucin synthesis and prostaglandin production in rats. Moreover, it significantly enhanced the levels of glutathione (GSH) and the activity of superoxide dismutase (SOD) in acetic acid-treated rats with gastric ulcers, while it reduced the amount of thiobarbituric acid reactive substances (TBARS).^133^

EMMPs also played a protective role in the gastric mucosa by strengthening the gastric immune barrier. *H. erinaceus* polysaccharides exerted a protective effect on the gastric mucosa by activating the repair and defense systems of the host and reducing the inflammatory cytokines IL-1β and TNF-α.^132^ Interestingly, although two polysaccharides, β-glucan H6PC20^43^ and α-heteropolysaccharide HPB-3,^42^ both have gastroprotective activity, the macromolecular β-glucan has better repair and defense system effects, while the small molecule α-heteropolysaccharide was specially anti-inflammatory,^132^ indicating that the difference in gastroprotective activity of polysaccharides may be related to their structures. *F. velutipes* polysaccharides (FVPs) alleviated ethanol-induced injury in GES-1 cells by increasing the gastric mucosal blood flow and promoting the defense function of the gastric mucosa with upregulation of the mRNA expression of triglyceride factor 2 (TFF2), prostaglandin E2 (PGE2), and epidermal growth factor (EGF).^134^

### Impact of EMMPs on Gastritis and *Helicobacter pylori*

Gastritis could be classified into different types based on its etiology including *H. pylori*-related gastritis, stress-related gastritis, autoimmune gastritis, drug-induced gastritis, reflux gastritis, and others. Three processes of the pathological changes in gastritis caused by different etiologies usually involve epithelial damage, mucosal inflammation response, and epithelial regeneration.^147^ Experiments using a chronic atrophic gastritis cell model showed that polysaccharides isolated from *H. erinaceus* significantly inhibited the growth of precancerous MC cells transformed by MNNG cells and interfered with MC cells through inducing cell cycle arrest,^41^ suggesting that EMMPs had possible antigastritis activity (Table 2).

The gastric microbiota, which constitute the biological barrier, help maintain gastric homeostasis. Gastritis is typically caused by inflammation of the gastric mucosa, resulting in changes in gastric acid secretion and pH values and affecting the abundance of gastric microbiota. Previous research demonstrated that such alterations could result in the overgrowth of harmful bacteria, thereby damaging gastric mucosal health.^148^ The abundance of gastric microbiota in patients with chronic atrophic gastritis was significantly lower than in those with chronic non-atrophic gastritis. Additionally, in chronic gastritis patients infected with *H. pylori*, the abundance of beneficial bacteria such as *Aliidiomarina*, *Reyranella*, *Halomonas*, *Pseudomonas*, and *Acidaminococcus* in the gastric microbiota was lower.^149^ This indicated that *H. pylori* infection altered the gastric microbiota and reduced the microbial diversity of the gastric mucosa. Zhu et al. found that *H. erinaceus* polysaccharide extract has anti-*H. pylori* activity.^135^ Besides, the ethanol extracts from *H. erinaceus*, *G. lucidum*, *Pleurotus eryngii*, *P. ostreatus*, *L. edodes*, and 14 other types of mushrooms inhibited the growth of *H. pylori*, with minimum inhibitory concentration (MIC) values less than 3 mg/mL.^150^ Unfortunately, studies investigating the important role of gastric microbiota with EMMPs against *H. pylori* are still rare. Such a future investigation would help to elucidate the possible synergistic action between EMMPs and gastric microbiota against *H. pylori*.

### Effect of EMMPs on Gastric Ulcers

Gastric ulcers are a common digestive tract ulcer disease primarily caused by a reduction in the gastric mucosal defense ability, leading to an increase in aggressive factors causing injury and the subsequent formation of ulcers. EMMPs could alleviate gastric ulcers possibly through promoting the release of gastric defensive factors, regulating gastric fluid secretion, suppressing inflammation, and improving antioxidant status (Table 2).

Mucus interacts with defensive factors such as NO, PGE2, and EGF to sustain the integrity and form the primary defense of the gastric mucosa.^137,151^ In animal models of gastric ulcers, the contents of PGE2, EGF, vascular endothelial growth factor (VEGF), basic fibroblast growth factor (bFGF), and NO in gastric tissue were significantly reduced. Polysaccharides from *Termitomyces eurrhizus*, *H. erinaceus*, and *G. lucidum* could reverse these changes.^147,149,151,160^ Besides, the expression of cyclooxygenases (COX) 1 and 2 was reduced during the peak period of gastric ulcers, which is related to the reduction of mucosal PGE2 synthesis. *T. eurrhizus* polysaccharide can effectively increase the synthesis of PGE2 by modulating the expression of COX 1 and 2.^137^*G. lucidum* polysaccharides may increase prostaglandin concentration and gastric mucus levels in the stomachs of rats, thereby accelerating the healing of gastric ulcers.^143^

Excessive gastric acid secretion will stimulate the production of pepsin, alter the permeability of the gastric mucosal wall, and facilitate ulcers, while gastrin and histamine are the stimulators of gastric acid secretion.^152^ EMMPs have a role in regulating gastric fluid secretion and strengthening the gastric chemical barrier. *H. erinaceus* polysaccharides decreased the ulcerated area in ethanol-induced ulcers in mice.^138^ It was found that *H. erinaceus* polysaccharides reduced ethanol-induced gastric mucosal lesions and pyloric ligation-caused gastric ulcers. Specifically, these polysaccharides modulated the volume of gastric secretions (gastric fluid, gastric acid, pepsin, and gastrin) in rats with ulcers.^141^

Damage to the gastric mucosa activates the inflammatory process, thereby increasing the secretion of inflammatory cytokines, such as TNF-α, IL-1β, and IL-6.^153^ Several studies showed that polysaccharides from *G. lucidum* and *H. erinaceus* markedly reduced the levels of TNF-α, IL-1β, and IL-6.^146,149,151^ Mechanistically, the NLRP3 inflammasome is activated under gastric ulcer conditions, and its activation can be triggered through the NF-κB pathway, thereby triggering increased secretion of inflammatory cytokines.^154^ Water extract of *Amauroderma rugosum* containing polysaccharides could reduce the ulcer area in the gastric ulcer models and inhibit gastric inflammation by suppressing the NF-κB/NLRP3 pathway.^140^ In addition, myeloperoxidase (MPO) activity is commonly used to evaluate leukocyte infiltration and inflammatory dysfunction.^155^*T. eurrhizus* and *G. lucidum* polysaccharides could significantly decrease MPO activity,^137,142^ indicating the efficacy of reducing inflammatory response of EMMPs.

After the gastric mucosa is stimulated by exogenous factors, the overexpression of reactive oxygen species can induce oxidative damage to the tissue, thereby promoting the formation of ulcers.^156^ SOD can scavenge free radicals in the body, thereby enhancing the antioxidant status. More research indicated that EMMPs alleviated gastric ulcers by improving the antioxidant status. The polysaccharides from *H. erinaceus* and *G. lucidum* exhibited the clearance of 2,2-diphenyl-1-picrylhydrazyl and hydroxyl radicals, along with enhanced SOD activity and thus improved the antioxidant status of gastric tissue.^139,141,142^ Interestingly, the gastroprotective effects of the *G. lucidum* polysaccharides (GLPs) with different molecular weights (100 kDa, 10 kDa, and 1 kDa) on ethanol-induced acute gastric injury in rats were evaluated, and the results showed that GLPs above 10 kDa have the best effects, which demonstrated the influence of different structures of polysaccharides on their activities.^142^

### Effect of EMMPs on Gastric Cancer

The pathogenesis of gastric cancer remains not entirely clear. Reports indicated that the causative factors, including the presence of *H. pylori*, genetic factors, alcohol consumption, obesity, and gastroesophageal reflux disease, contributed to an increased incidence of gastric cancer.^157^ Gastric cancer also impacted the gastric microbiota. In *H. pylori*-negative gastric cancer patients, an enrichment of Proteobacteria and Firmicutes was observed. However, in *H. pylori*-positive gastric cancer patients, *Neisseria* was the only identified enriched genus in the Betaproteobacteria class.^158^ Currently, surgery, radiotherapy, chemotherapy, and immunotherapy are used for the treatment of gastric cancer. However, these treatments exhibit high toxicity to the normal cells of the nervous system and gastrointestinal tract and have not achieved satisfactory therapeutic effects.^159^ Existing experiments suggest that EMMPs play a positive role in gastric cancer therapy (Table 2).

A study regarding the anticancer effects of *G. lucidum* polysaccharides on the gastric cancer cell line AGS indicated that the polysaccharides significantly induced cells apoptosis and autophagy and downregulated the expression levels of Bcl-2 and caspase-3 while they upregulated the cleavage of PAR.^160^ The selenium-enriched *P. ostreatus* polysaccharides reduced viability, induced apoptosis, inhibited migration and invasion, disrupted the Bax/Bcl-2 ratio, and suppressed the epithelial-to-mesenchymal transition in human gastric cancer MGC-803 cells.^161^ Polysaccharides isolated from the *P. ostreatus* mycelium significantly reduced colony formation and invasion ability in gastric cancer BGC-823 cells and reduced tumor weight and volume and exhibited antitumor effects against cancer in mice implanted with BGC-823 cells *in vivo*.^144^ Polysaccharide EP-1 isolated from *H. erinaceus* induced cell apoptosis and cell cycle arrest, and modulated the expression of Bax, Bcl-2, and caspase-3 in precancerous human gastric cells.^145^ A meta-analysis of 8009 patients from randomized controlled trials indicated that the immunopotentiator polysaccharide K from *Coriolus versicolor* added to standard chemotherapy increased the survival of patients after curative gastric cancer resection over chemotherapy alone.^162^ Besides, another clinical study also showed that chemo-immunotherapy using lentinan prolongs the survival of patients with advanced gastric cancer compared to chemotherapy alone.^163^

### Mechanisms of Action of EMMPs against Gastric Diseases

**Figure 3:**
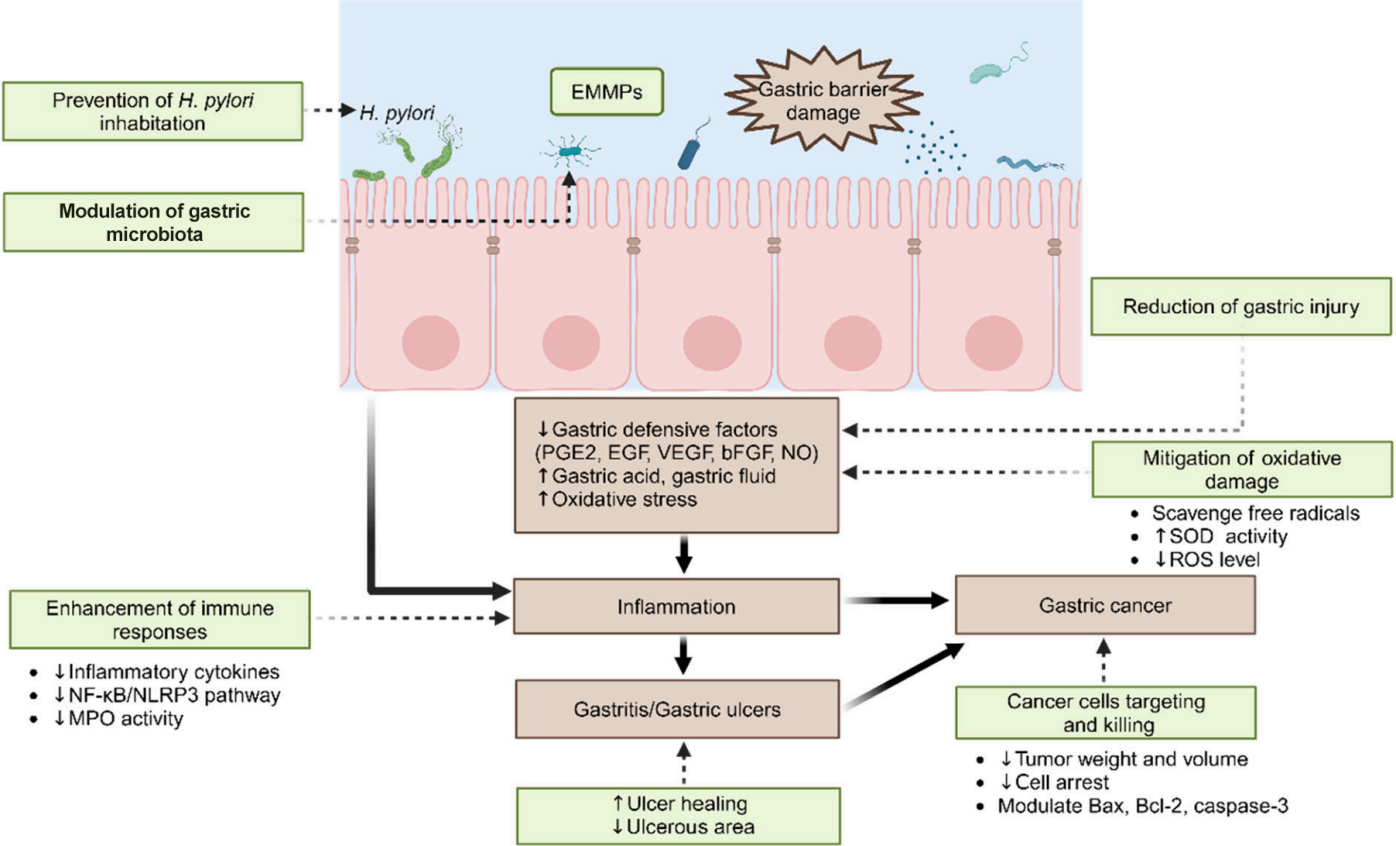
Mechanism of action of EMMPs in protecting gastric health. Abbreviations: EMMPs, edible and medicinal mushroom polysaccharides; *H. pylori*, *Helicobacter pylori*; PGE2, prostaglandin E2; EGF, epidermal growth factor; VEGF, vascular endothelial growth factor; bFGF, basic fibroblast growth factor; NO, nitric oxide; SOD, superoxide dismutase; ROS, reactive oxygen species; NF-κB, nuclear factor-kappa B; NLRP3, NOD-like receptor family pyrin domain containing 3; MPO, myeloperoxidase; Bax, BCL-2-associated X protein; Bcl-2, B-cell lymphoma-2.

Through the above summary and analysis, it was concluded that the EMMPs could be used as potential gastroprotective agents to protect the gastric barrier against damage and alleviate the subsequent diseases including gastritis, gastric ulcers, and even gastric cancer. In summary, EMMPs can serve as prebiotics, preventing *H. pylori* from adhering, colonizing, and invading the gastric cell wall, modulating the gastric microbiota, promoting the release of gastric defensive factors, regulating gastric fluid secretion, improving antioxidant status, modulating immune response, and targeting and killing gastric cancer cells. Figure 3 summarizes the mechanism of action of EMMPs in protecting gastric health.

## Intestinal Protective Effects and Possible Mechanisms of EMMPs

Damage to the intestinal mucosal barrier is strongly related to the development of many intestinal inflammatory diseases. It not only affects the selective absorption of water and nutrients in the digestive process but also allows bacteria and toxins in the intestinal tract to cross the mucosal barrier and flow into the portal vein and lymphatic system to arouse bacterial translocation. Causes of intestinal mucosal barrier dysfunction, such as chronic inflammation, bacterial dysbiosis, and environment changes, can independently or synergistically cause biochemical cascade reactions, leading to chronic inflammation, which can further cause a variety of intestinal diseases, for example, IBD, colorectal cancer (CRC), and intestinal infection.

Numerous studies have confirmed that EMMPs play an important role in regulating intestinal health as manifested primarily in the following three aspects: (1) regulating the intestinal microbiota and maintaining intestinal homeostasis; (2) improving inflammatory bowel disease; (3) alleviating colorectal cancer; and (4) inhibiting the growth of pathogenic microorganisms. Table 3 summarizes the intestinal protective effects and mechanisms of EMMPs.

**Table 3:** Intestinal Protective Activities and Associated Mechanisms of EMMPs^a^

Polysaccharides	Source	Subject	Treatment	Mechanism and effect	ref
Intestinal Homeostasis					
Consisted of 48.45% glucose, 15.40% mannose, 14.60% xylose, 11.80% fucose, and 9.90% galactose	*F. velutipes*	*In vitro* gastrointestinal simulation and *in vitro* human fecal fermentation	Added to the media at a ratio of 1:10 (w/v)	↑Bifidobacteriaceae, Bacteroidaceae; ↓Lachnospiraceae, Enterococcaceae; ↑SCFAs	(164)
Consisted of glucosamine and glucose in a molar ratio of 67:33 with degree of acetylation of 61.91% and crystallinity index of 25.40%	*C. comatus*	*In vitro* human fecal fermentation	NA	↑*Bacteroides*, *Bifidobacterium*; ↑SCFAs (e.g., propionic and butyric)	(165)
Molecular ratios: Man 1.00, GlcA 0.07–0.09, Glc 0.02–0.03, Xyl 0.30–0.32 and Fuc 0.19–0.20, MW: 18.6 × 10^5^ Da	*T. fuciformis*	*In vitro* gastrointestinal simulation and *in vitro* human fecal fermentation	Added to the media at a ratio of 1:10 (w/v)	↑*Phascolarctobacterium*, *Lachnoclostridium*; ↑SCFAs	(166)
Composed of Glc 62.04%, Gal 19.50%, Man 8.17%, Xyl 3.23%, Fuc 3.00%, and Ara 2.46%; Mw: 3.62 × 10^6^Da	*A. bisporus*	*In vitro* GIT simulation and *in vitro* human fecal fermentation	NA	↑SCFAs; ↑*Prevotella*, *Phascolarctobacterium*, and *Parabacteroides*; ↓*Fusobacterium*, *Escherichia*, *Sutterella*, and *Desulfovibrio*	(167)
Four polysaccharide extracts (88–93% of polysaccharides; molecular ratios: Gal 1.73–1.86, Glc 0.71–0.75, Man 0.71–0.72, GlcA 0.71–0.73; Mw: 123.6–135.8 kDa)	*V. volvacea*	*In vitro* GIT simulation and *in vitro* human fecal fermentation	NA	↑Bacteroidetes/Firmicutes ratio; ↑*Bacteroides* and *Phascolarctobacterium*; ↓*Escherichia*–*Shigella*; ↑SCFAs (acetic, propionic, and butyric)	(168)
93.27% Purity	*Lentinula*	LPS induced inflammatory response in intestine, juvenile taimen	5.00, 10.00 g/kg for diets	↑Relative abundance of beneficial bacteria (such as Lactobacillaceae, Lachnospiraceae, and Ruminococcaceae); ↓Relative abundance of detrimental bacteria (such as Enterobacteriaceae, Fusobacteriaceae, and Flavobacteriaceae); ↓Expression levels of inflammatory factors	(169)
HECP and HERP with similar structure, and the main monosaccharides were Glc and Gal	*H. erinaceus*	Healthy mice	100, 200, 400 mg/kg	↑SCFAs in colonic and cecum contents; ↓pH value of the intestine; improved colonic health	(170)
Purity 91.25 ± 3.14%, composed of 79.11% Glc, 10.37% Gal, 5.75% Man and 1.77% Xyl; Mw: 426 kDa	*P. eryngii*	Healthy mice	0.2, 0.4, 0.8 g/kg	↑SCFAs in cecum and colon contents; ↓pH value of the intestine; ↑Moisture contents of feces and bowel movements; ↑Relative abundance of beneficial bacteria (Porphyromonadaceae, Rikenellaceae, Bacteroidaceae, and Lactobacillaceae); ↓Relative abundance of detrimental bacteria (Lachnospiraceae and Ruminococcaceae)	(171)
Consisted of glucosamine, glucuronic acid, glucose, xylose, arabinose, and fucose with the molar ratio of 0.9:7.6:44.2:7.0:35.8:4.5, with β type glycosidic bonds	*A. auricular*	Healthy mice	40, 80, 160 mg/kg	↑SCFA concentrations; ↓pH value of the intestine; ↓Firmicutes/Bacteroidetes ratio; ↑relative abundances of Porphyromonadaceae and Bacteroidaceae	(172)
Consisted of mannose, glucose, xylose, arabinose and fucose with the molar ratio of 6.6:27.8:18:1.5:5.2, with nonstarch polysaccharides with β-type glycosidic bonds	*F. velutipes*	Healthy mice	40, 80, 160 mg/kg	↑SCFA concentrations; ↓pH value of the intestine; ↓Firmicutes/Bacteroidetes ratio, Relative abundances of *Lactobacillus* and *Akkermansia*; ↑Relative abundances of Porphyromonadaceae and Bacteroidaceae	(173)
30% purity	*G lucidum* and *Poria cocos*	Healthy C57BL/6J mice	750 mg/kg	↑Beneficial bacteria (e.g., *Bifidobacterium choerinum* and *Eubacterium rectale*); ↓Pathogenic bacteria; ↑SCFAs and lactic acid	(174)
FVP1, composed of mannose (7.74%), glucose (70.41%), and galactose (16.38%); Mw: 54.78 kDa	*F. velutipes*	Healthy C57BL/6 mice	20, 40, 80 mg/kg	↑Villus height and V/C value; ↑Firmicutes; ↓Bacteroidetes; Improved gut health	(175)
WS and WI polysaccharide extracts (composed of WS: Xyl, Gal, Glc, and Man; and WI: Glc)	*Dictyophora indusiata*	Young mice	200 mg/kg	↑Intestinal microbial diversity; ↑*Lactobacillus*; ↑SCFAs (e.g., acetic and butyric)	(176)
Inflammatory Bowel Disease					
NA	*P. cocos*	TNBS-induced IBD, mice	100, 300 mg/kg by gavage	↓Mortality; ↓Disease activity index; ↓Proinflammatory cytokine levels in colon tissue and serum; ↑Anti-inflammatory cytokine levels	(177)
wHEP-1 composed of mannose, glucose, and galactose in a molar ratio of 1.2:16.9:1.1; wHEP-2 and wHEP-3 composed of glucose and galactose in different molar ratios. wHEP-1 Mw: 5010 Da; wHEP-2 Mw: 1812; wHEP-3 Mw: 1118 Da	*H. erinaceus*	Acetic acid enema induced UC, rats	1.2 g/kg	All showed good anti-inflammatory activity, with wHEP-1 performing best	(178)
Primarily comprised glucose (99.23%)	*G. applanatum*	DSS-induced colitis, mice	250, 500 mg/kg	↓Mortality, body weight, Disease Activity Index score, colon length, and histologic score; Restoration of the intestinal barrier and regulation of intestinal flora; ↓Abundance of *E. coli*, *Shigella*, enterococci, and staphylococci	(179)
Polysaccharopeptide, with a mass ratio of 4.87:1 of polysaccharide to peptide	*Sanghuangporus lonicericola*	DSS-induced UC, mice	200 mg/kg	↓Weight loss; ↓Proinflammatory cytokine gene expression levels in the colon; ↑Level of anti-inflammatory cytokine gene expression in the colon	(180)
β-glucan, consisted of fucose (0.025), glucosamine hydrochloride (0.004), galactose (0.063), glucose (0.869) and mannose (0.038)	*G. frondosa*	OXZ-induced UC, mice	80, 160, 320 mg/kg	↑Colon length; ↑Leukocyte, platelet, and neutrophil levels; ↓Proinflammatory cytokines	(181)
Composed of mannose (6.5%), glucose (32.38%), and galactose (52.56%), Mw: 4.6 kDa	*H. erinaceus*	Non-artificially induced UC in cynomolgus monkeys	500 mg/kg	↑Nutritional status; ↓Incidence of diarrhea and inflammation	(182)
IOP, included Man, Rha, Glc, Gal, Xyl, and Ara (molar ratio: 9.2:4.4:46.6:11.5:11.1:4.3)	*I. obliquus*	DSS-induced colitis, mice	100, 200, 300 mg/kg	↓Disease activity index, pathologic changes; ↑TJ proteins occludin and ZO-1 in colon tissue; ↑Th1/Th2, Th17/Treg homeostasis	(183)
FMPS, with (1→3)-linked β-d-glucopyranosyl backbone	Huangshan floral mushroom	DSS-induced colitis, mice	50, 200 mg/kg	↑Expression of occludin, ZO-1, and Muc-2; ↓Secretion of proinflammatory cytokines; ↑SCFAs; Restoration of intestinal Th17/Treg balance; Regulation of gut microbes	(184)
Consisted of mannose, xylose, caramel, glucose, and glucuronic acid; Mw: 5.8 × 10^3^ kDa	*T. fuciformis*	DSS-induced UC, mice; LPS-stimulated Caco-2 cells	50, 100, 200 mg/kg	Improved symptoms of weight loss; ↑Colon length in mice; ↓Inflammatory cell infiltration and restoration of intestinal epithelial barrier integrity in mice; ↓Expression of proinflammatory cytokines in Caco-2 cells; ↑Expression of intestinal barrier and mucus barrier proteins in Caco-2 cells	(185)
HFP, polysaccharide content 86.61 ± 5.26%; composed of glucose, galacturonic acid, mannose and fucose (31.37:3.83:1.07:1), Mw: 4.783 × 10^3^ Da	*H. caput-medusae*	DSS-induced colitis, mice	400 mg/kg	↑Body weight, colon length, and mucus layer thickness; ↓Diarrhea; ↓Intestinal transit rate and improved expression of TJs	(186)
EPA-1, consisted of Man, Glc, and Gal in a molar ratio of 2.2:1.0:3.2, Mw: 9.97 × 10^4^ Da	*P. eryngii*	LPS treated RAW 264.7 macrophages	25, 50, 100 μg/mL	↑signal protein of p38, ERK, JNK in MAPKs and translocation of nuclear NF-κΒ	(48)
FVP, Mw 15 961 Da; Fermented FVP (FFVP), Mw 15,702 Da	*F. velutipes*	LPS-induced intestinal inflammation, mice	50, 100 mg/kg	↑Antioxidant capacities; ↓Secretion and mRNA expression of IL-1β, IL-6, IL-18, and TNF-α	(187)
Mw: 86.67 kDa	*H. erinaceus*	DSS-induced colitis, mice	150, 250, 500 mg/kg	Exerted antioxidant and anti-inflammatory effects; ↓Phosphorylation of NF-κB signaling pathway related proteins; Regulated intestinal flora; Maintained the integrity of the intestinal barrier	(13)
Mw: 9.9 kDa	*H. erinaceus*	DSS-induced colitis, mice	50, 100, 200 mg/kg	↓Production of proinflammatory cytokines and COX-2 in the colon; ↓Activation of the NF-κB signaling pathway; ↑Relative abundance of *Akkermansia muciniphila* and regulation of gut microbes	(188)
FVP1, composed of glucose (56.2%), mannose (29.7%) and galactose (14.1%), Mw: 54.78 kDa	*F. velutipes*	DSS-induced colitis, rats	50, 100, 200 mg/kg	↓TLR4/NF-κB signaling pathway; ↓Proinflammatory cytokines; ↑Anti-inflammatory cytokines; ↑Ratio of Firmicutes/Bacteroidetes	(189)
Composed of fucose, galactose, glucose, and mannose (11.59%, 36.28%, 31.60%, and 20.37%, respectively), contained a small amount of GalA, GulA, and GlcA; Mw: 17.249 kDa	*B. aereus*	DSS-induced colitis, mice	200, 400 mg/kg	↑Expression of intestinal mucosal and TJ proteins; Restored the compromised mucus barrier; ↓Activation of inflammatory signaling; Reshaped the gut microbiota; ↓Cytokine levels through the MANF-BATF2 signaling pathway	(190)
Polysaccharide content 74%, composed of glucose, mannose, and galactose, with small amounts of arabinose, rhamnose, and ribose; Mw: 32.9 kDa	*P. sanguineus*	DSS-induced colitis, mice	100, 200, 400 mg/kg	↓Decreased disease activity index; ↑Colon length; ↓Serum LPS; ↑Expression of TJ proteins ↓Proportion of Th cells; ↓MPO activity	(191)
CSP, with backbone 1,4-glucose and 1,4-galactose, Mw: 28 kDa	*C. sinensis*	Cyclophosphamide (Cy)-induced intestinal mucosal immunosuppression and microbial dysbiosis, mice	25, 50, 100 mg/kg	↑TLRs (TLR-2, TLR-4, TLR-6) and NF-κB pathway key proteins (p-IκB-α, NF-κB p65); ↑SCFAs; Promoted Th cell differentiation; ↑Diversity of microbial communities	(192)
DIP, total sugar content: 97.6%, a homogeneous β-(1→3)-d-glucan with side branches of β-(1→6)-glucosyl units; Mw: 536 kDa	*D. indusiata*	DSS-induced colitis, mice	25, 50, 100 mg/kg	Restored intestinal barrier function; ↓M1 macrophage polarization; ↑M2 macrophage polarization; ↓Oxidative stress and inflammation; Inhibited key signaling pathways associated with colitis	(193)
Purity 94.46%	*A. blazei* Murrill	DSS-induced colitis, mice	200 mg/kg	↓Weight loss, colon shortening, and disease activity index scores; ↑TJ and mucus content; ↓Inflammation and oxidative stress	(194)
PTRP, composed of 0.93% fucose, 7.56% galactose, 76.25% glucose, 14.25% mannose, 0.55% ribose and 0.46% glucuronic acid; Mw: 4.50 × 10^6^ Da	*P. tuber-regium*	DSS-induced colitis, mice	100, 200 mg/kg	Improved the clinical symptoms and intestinal tissue damage caused by colitis; ↓Secretion of proinflammatory cytokines and myeloperoxidase activity; ↓Levels of oxidative stress factors; ↑Antioxidant capacity and the production of SCFAs; ↑Diversity and abundance of beneficial bacteria	(195)
Contents were 65.19% ± 2.52% carbohydrate, 6.24% ± 0.59% polyphenol, 15.04% ± 3.21% uronic acid, and 5.33% ± 0.37% protein	*H. erinaceus*	DSS-induced UC, mice	200, 300, 400 mg/kg	↓Oxidative damage; ↓MDA levels; ↑Levels of the antioxidant enzymes SOD and CAT in colon; ↓Levels of the proinflammatory factor; ↑Anti-inflammatory factor; ↓levels of NLRP3, ASC, and caspase 1 P20; ↑Abundance of gut microbiota	(196)
WPEP, β-type glycosidic linkages and mainly composed of xylose, mannose, glucose, and galactose with a molecular ratio of 21.35:3.28:73.22:1.63; Mw: 167 kDa	*P. eryngii*	DSS-induced colitis, mice	0, 0.2, 0.8 g/kg	↓Disease Activity Index; ↑Colon length; ↓Proinflammatory cytokine concentrations and proinflammatory protein expression; ↓Inflammation; ↓Accumulation of immune cells, macrophages, and neutrophils; ↑Intestinal flora structure and quantity	(197)
Consisted of Glc, Gal, Man, Ara, Fuc, and Xyl in molar ratio 14.368:2.594:1.956:1.552:1.466:1, its backbone consisted of α1→3-d-Glc and α1→6-d-Glc; MW: 2.8 × 10^4^ Da	*P. linteus*	DSS-induced UC, mice	300, 600 mg/kg	↓UC symptoms (weight loss, reduced food intake, increased disease activity index); Ameliorated histopathological colon tissue damage; ↓Levels of proinflammatory factors and oxidative stress-related enzymes iNOS and MPO; ↑Anti-inflammatory factor and expression of TJs; Restored gut microbiota diversity and species abundances; ↑SCFAs	(198)
FVP, with β-glycosidic bonds and a furanose ring, composed of 7473.14 kDa (48.09%) and 15.077 kDa (51.91%) components, linked by mannose, glucose, xylose, arabinose, and fucose	*F. velutipes*	DSS-induced colitis, mice	400 mg/kg	↑Capacity of metabolic and biogenesis; ↓Symptoms of UC; ↓Proinflammatory cytokines; ↑Microbial community diversity; ↓Ratio of Firmicutes/Bacteroidetes	(199)
Aap, composed of 74.04% sugar, consisted of rhamnose, mannose, and glucose (1.46:2.34:0.63), and contained a few fucose, xylose, and galactose	*A. auricula-judae* (Bull.)	DSS-induced colitis, mice	40 mg/kg	↓Serum inflammatory cytokine level; ↓Weight loss, colon damage, mucosal inflammation, and impairment of intestinal barrier function; ↑Bacteroidetes; ↓Firmicutes, *Ruminococcus*, Deferribacteres, and Actinobacteria	(200)
TPs, Mw 246–305 kDa, and comprised mannose, glucuronic acid, xylose, fucose, glucose, galactose, arabinose, rhamnose, and ribose	*T. fuciformis*	DSS-induced colitis, mice	200, 300 mg/kg	↓Colon shortening and colonic tissue damage in mice; ↑Anti-inflammatory cytokines; ↓Proinflammatory cytokines; ↑Intestinal community diversity; ↑Relative abundance of *Lactobacillus*, *Odoribacter*, *Helicobacter*, Ruminococcaceae, and Marinifilaceae; Influenced tyrosine biosynthesis, tryptophan metabolism, and bile acid metabolism	(201)
GLP, composed of β-glucan (>90%) that contained a 1,6-linked β-d-Glcp backbone with different length branches consisting of terminal and 1,4-linked Glcp residues attached to 0–4 alternating Glc residues on the backbone	*G. lucidum*	DSS-induced colitis, rats	393.75 g/kg by oral feeding	↓Inflammatory response and colon cancer risk; ↓Number of pathogens (*E. coli*, *Shigella*); ↑SCFAs; ↑Immunity of the colon	(202)
EP-1, with carbohydrate content 95.7%, Mw: 3.1 kDa	*H. erinaceus*	Acetic acid-induced UC, rats	0.6, 1.2 g/kg	↑Diversity and abundance of gut microorganisms; ↑SCFAs such as acetic acid and butyric acid; ↓Secretion of proinflammatory factors; ↓GPR41/43 expression	(203)
Colorectal Cancer					
89% carbohydrate, mainly consisted of α-polysaccharides, composed of arabinose, galactose, glucose and cellose at the molar ratio of 11:3:3:1	*G. lucidum*	Human colon cancer cells LoVo	2.5, 5, 10 mg/mL	↓Migration; ↑Apoptosis; ↑DNA fragmentation, morphological alterations and lactate dehydrogenase release; ↑Expression of Fas and caspase-3 proteins; ↓Expression of cleaved poly(ADP-ribose) polymerase	(204)
NA	*G. lucidum*	Human colon cancer cell lines HT-29 and HCT116; HT-29 cell xenograft cancer, mice	0, 2.5, 5, 10 mg/mL; 150, 300 mg/kg	*In vitro*: Induced the initiation of autophagy of cancer cells with ↑level of LC3-II protein, GFP-LC3 puncta; Blocked autophagic flux; ↑MAPK/ERK activation. *In vivo*: ↓tumor growth and autophagic flux	(205)
Composed of arabinose, galactose, glucose, and mannose at a molar ratio of 8.99:11.15:1.2:1.97	*H. erinaceus*	Human colorectal cancer cells HCT-116 and DLD1	0.3, 0.6 mg/mL	↓Growth of colorectal cancer cells; Induced apoptosis via the caspase-9-dependent intrinsic mitochondrial pathway; ↑Level of ROS; Modulated Bax and Bcl-2 expression	(206)
Consisted of fucose, galactose, glucose, and mannose in molar ratio of 11.81:22.82:44.28:21.09	*H. erinaceus*	Human colorectal cancer cells HCT-116	0.15, 0.3 mg/mL	↓Growth of colon cancer cells; Arrested the cell cycle at the S-phase	(207)
Lentinan, Mw: 507.2 kDa, composed entirely of d-glucose; Mw: 3.252 × 10^4^ Da	*L. edodes*	Human colon cancer cells HT-29; HT-29 cell xenograft cancer, mice	800, 1600 μg/mL; 1, 5 mg/kg	*In vitro*: ↓Tumor growth; Induced autophagy and endoplasmic reticulum stress. *In vivo*: ↓Solid tumors; Activated Ca^2+^-induced cell death; Induced autophagy and endoplasmic reticulum stress	(208)
Lentinan	*L. edodes*	Human colon cancer cell line HT-29	0, 500, 1000 μg/mL	↓Cell proliferation, wound healing, and colony formation abilities and migration; ↑phospho-p38; ↓phopho-ERK1/2; ↑ROS production	(209)
30% purity	*G. lucidum*	Colorectal cancer, (Apc^Min/+^) mice	750 mg/kg	Shifted colonic M1 to M2 macrophages; ↑Anti-inflammatory cytokines; ↑Relative abundance of Bacteroidetes, polysaccharide-degrading bacteria, and SCFA-producing bacteria; ↓Relative abundance of harmful bacteria	(210)
Composed of rhamnose, xylose, mannose, and glucose, with a molar ratio of 1.00:1.04:1.11:6.21; Mw:155 kDa	*G. frondosa*	AOM/DSS-induced colon cancer, mice	125, 250, 500 mg/kg	↓Colon MPO activity and disease activity index; ↓NO; ↑GSH and SOD; ↓Number and volume of tumors; ↓Proportion of stool blood and anal abscission; ↓Proinflammatory cytokines COX-2, IL-6, TNF-α; Regulated the Wnt/β-catenin/GSK-3β signaling pathway	(211)
NA	*L. edodes*	CT-26 tumor cells xenograft cancer, mice	30 mg/kg	↓lymphangiogenesis and lymphatic metastasis; ↓secretion of VEGF-C by cancer associated fibroblasts; Regulated lymphangiogenesis via TLR4/JNK signaling pathway	(212)
Composed of mannose, rhamnose, glucose, galactose, xylose, and arabinose (molar ratio: 9.2:4.4:46.6:11.5:11.1:4.3)	*I. obliquus*	AOM/DSS-induced colorectal cancer, mice	150 mg/kg	↑Expression of the NLRP3 inflammasome, IL-1β, and IL-18 in the colon; ↓Body weight loss, colon tissue damage, colon shortening, and expression of proinflammatory mediators	(213)
SVP-A-1, Mw: 22.5 kDa	*S. vaninii*	Human colon adenocarcinoma cells SW480 and Caco-2; Colon cancer, Apc^Min /+^ mice	0–8 mg/mL; 500 mg/kg	*In vitro*: ↓Cell viability, migration capacity, and cell proliferation. *In vivo*: ↓Number of tumors in the colorectum of mice; ↓Angiogenesis in tumor tissue; Activated Th1 immunity in mice; ↓Intestinal dysbiosis	(214)
β-1,3-glucan	*G. lucidum*	AOM/DSS-induced colorectal cancer, mice	393.75 g/kg	↑Colon length; ↓30% of mortality rate; ↓Relative abundance of cecal *Oscillospira* and an unknown genus of Desulfovibrionaceae; Improvement of microbiota dysbiosis	(215)
Total carbohydrate content 89.32%; composed of arabinose (4.19%), mannose (15.69%), glucose (78.15%), and galactose (1.97%); Mw: 25.0 kDa	*G. lucidum*	AOM/DSS-induced colorectal cancer, mice	200, 300 mg/kg	↓Disease activity index score and total number and size of tumors; Ameliorated microbiota dysbiosis; ↑Gut barrier function and SCFA production; Alleviated endotoxemia by inhibiting TLR4/MyD88/NF-κB signaling	(216)
Enteropathogenic Bacteria					
PS-F2, heteropolysaccharides	*G. formosanum*	*L. monocytogenes* infected mice	50 mg/kg	Stimulated macrophages to initiate an inflammatory response, produce NO, and become more phagocytic	(217)
GpEPS, total polysaccharide 241.8 ± 2 mg/g	*G. applanatum*	*S. aureus* (ATCC 25923)	1 mg/mL	Showed antibacterial properties against the *S. aureus* with inhibition zone of 17.9 mm and MIC value of 1 mg/mL	(218)
BPP, contained neutral sugars glucose, galactose, rhamnose, fucose, mannose, and xylose and basic amino sugars glucosamine and galactosamine; Mw > 10000 Da	*L. edodes*	*Salmonella typhimurium* infected RAW 264.7 cells; *S. typhimurium* infected mice	*In vitro*: 1, 10, 100 μg/mL; *In vivo*: 10 mg/kg	*In vitro*: Altered morphology; ↑iNOS mRNA and protein expression *In vivo*: ↑Lifespan of mice; ↑Th1 cytokines: IL-1β, IL-2, IL-6, IL-12	(219)
PEPS, sulfated polysaccharides	*P. eryngii*	*S. aureus*, *E. coli*, and *L. monocytogenes*	10 mg/mL	Showed antibacterial effect	(220)
IPS, composed of rhamnose, inositol, and glucose with a molar ratio of 4.7:3.6:1	*G. frondosa*	*E. coli*, *S. aureus*, *B. megaterium*, and *L. monocytogenes*	10, 20 mg/mL	Showed antibacterial effect	(221)
Crude polysaccharides, 76.12% contents	*A. auricula-judae*	*E. coli* and *S. aureus*	NA	Showed antibacterial effect	(222)

a Abbreviations: SCFAs, short chain fatty acids; NA, not applicable; Man, mannose; GlcA, glucuronic acid; Glc/Glcp, glucose; Xyl, xylose; Fuc, fucose; Gal, galactose; Ara, arabinose; GIT, gastrointestinal tract; LPS, lipopolysaccharide; TNBS, 2,4,6-trinitrobenzenesulfonic acid; IBD, inflammatory bowel disease; UC, ulcerative colitis; DSS, dextran sulfate sodium salt; *E. coli*, *Escherichia coli*; OXZ, oxazolone; Rha, rhamnose; TJs, tight junctions; ZO-1, zonula occludens-1; Th1/2/17, trihydrophobin 1/2/17; Treg, regulatory T cell; Muc-2, Mucin-2; ERK, extracellular regulated protein kinases; JNK, c-Jun N-terminal kinase; MAPK, mitogen-activated protein kinase; NF-κB, nuclear factor-kappa B; IL-1β/2/6/12/18, interleukin-1 beta/2/6/12/18; TNF-α, tumor necrosis factor-alpha; COX-2, cyclooxygenase-2; TLR4, Toll-like receptor 4; GalA, galactonic acid; GulA, gulosine; MANF, mesencephalic astrocyte derived neurotrophic factor; BATF2, basic leucine zipper transcriptional factor ATF like 2; MPO, myeloperoxidase; TLRs, Toll-like receptors; p-IκB-α, phospho-inhibitory subunit of NF-κBα; MDA, malondialdehyde; SOD, superoxide dismutase; CAT, catalase; NLRP3, NOD-like receptor family pyrin domain containing 3; ASC, apoptosis-associated speck-like protein containing a CARD; iNOS, inducible nitric oxide synthase; GPR41/43, G-protein coupled receptor 41/43; caspase-3/9, cysteine-containing aspartate-specific protease-3/9; ADP, adenosine diphosphate; LC3, microtubule-associated protein 1 light chain 3; GFP, green fluorescent protein; ROS, reactive oxygen species; Bax, BCL-2-associated X protein; Bcl-2, B-cell lymphoma-2; AOM, ammonia-oxidizing microorganisms; NO, nitric oxide; GSH, l-glutathione; Wnt, wingless/integrated; GSK-3β, glycogen synthase kinase-3β; VEGF-C, vascular endothelial growth factor-C; MyD88, myeloid differentiation factor 88; MIC, minimum inhibitory concentration; HECP, *H. erinaceus* crude polysaccharide; HERP, *H. erinaceus* refined polysaccharide; V/C, villus height/crypt depth; WS, Water-soluble; WI, water-insoluble.

### Role of EMMPs in Regulating Intestinal Microbiota and Maintaining Intestinal Homeostasis

Trillions of microorganisms, including at least 1000 different species of bacteria, live in the gut and are critical to human health. The intestinal microbiota have a harmonious and symbiotic relationship with the host. Once the balance is disturbed, the intestinal microecological system will be affected, subsequently resulting in intestinal disease. As nondigestible carbohydrates, EMMPs can reach the rectum and colon and act as prebiotics to influence the community composition and diversity of intestinal microorganisms (Table 3). After fermentation, EMMPs are converted to SCFAs by intestinal microbiota. SCFAs can reduce the luminal pH and exert properties such as regulating the immune system, inhibiting inflammation, and regulating colon cell permeability and intestinal barrier function.^20^ From *in vitro* fermentation models, polysaccharides in *F. velutipes* and *Coprinus comatus* were shown to regulate the abundance of intestinal bacteria, such as promoting the growth of Bifidobacteriaceae and Bacteroidetes and decreasing the number of Lachnospiraceae and Enterococcaceae at family levels.^164,165^ Likewise, polysaccharides from *Tremella fuciformis*, *A. bisporus*, and *Volvariella volvacea* raised the relative abundance of *Phascolarctobacterium*, and upregulated the amounts of SCFAs *in vitro*.^166−168^ In animal models, lentinan improved the relative abundance of beneficial bacteria (Lactobacillaceae, Lachnospiraceae, and Ruminococcaceae) and reduced that of harmful bacteria (such as Enterobacteriaceae, Fusobacteriaceae and Flavobacteriaceae) in the intestinal tract of juvenile taimen with intestinal dysbiosis.^169^ Both polysaccharides from *H. erinaceus* and *P. eryngii* could increase the content of SCFAs in the colon and cecum contents of mice and reduce intestinal pH, thus improving intestinal health.^170,171^ Polysaccharides from *A. auricula* and *F. velutipes* also had the similar effects in mice, and reduced the Firmicutes/Bacteroidetes ratio and increased the relative abundance of Porphyromonadaceae and Bacteroidetes.^172,173^ Besides, polysaccharides from *G. lucidum*, *F. velutipes*, and *Dictyophora indusiata* revealed the potential of prebiotics, effectively improved the tissue morphology of the small intestine, improved the structure of intestinal microbiota, and finally promoted intestinal health.^174−176^ In short, the supplementation of EMMPs can improve intestinal morphology, enhance intestinal mucosal barrier function, regulate the community abundance and structural composition of intestinal microorganisms, promote intestinal homeostasis, and have the potential to improve intestinal health.

### Attenuating Effects of EMMPs on IBD

**Figure 4:**
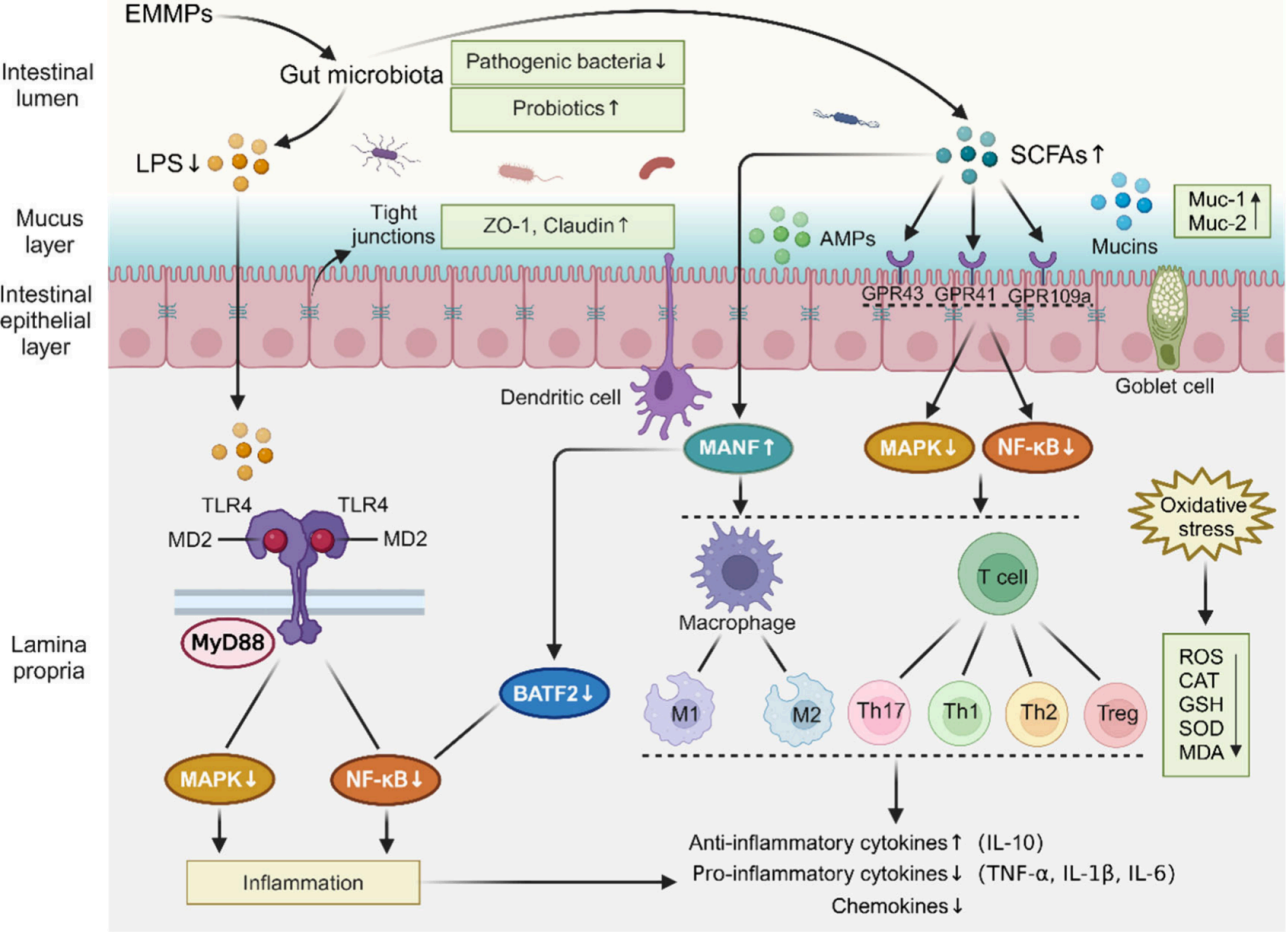
Mechanisms of EMMPs in alleviating IBD. Abbreviations: EMMPs, edible and medicinal mushroom polysaccharides; LPS, lipopolysaccharide; TLR4, Toll-like receptor 4; MD2, myeloid differential protein-2; MyD88, myeloid differentiation factor 88; MAPK, mitogen-activated protein kinase; NF-κB, nuclear factor-kappa B; ZO-1, zonula occludens-1; SCFAs, short chain fatty acids; AMPs, antimicrobial peptides; Muc-1/2, Mucin-1/2; GPR41/43/109a, G-protein coupled receptor 41/43/109a; MANF, mesencephalic astrocyte derived neurotrophic factor; BATF2, basic leucine zipper transcriptional factor ATF like 2; Th17/1/2, trihydrophobin 17/1/2; Treg, regulatory T cell; IL-6/10/1β, interleukin-6/10/1 beta; TNF-α, tumor necrosis factor-alpha; ROS, reactive oxygen species; CAT, catalase; GSH, l-glutathione; SOD, superoxide dismutase; MDA, malondialdehyde.

Crohn’s disease and ulcerative colitis are the two main types of IBD, characterized by increased intestinal permeability, chronic intestinal inflammation, and dysbiosis of the intestinal microbiota.^223^ EMMPs have been widely used in the inhibition of IBD and can increase body weight and colon length, and improve the colitis activity index and histological score of mice (Table 3).^177,178,180−182^ EMMPs usually alleviate IBD by restoring the intestinal barrier (physical and chemical barriers), modulating the intestinal immune system and anti-inflammatory effects (immune barriers), and regulating the diversity and abundance of microbiota (biological barriers). The corresponding mechanisms of EMMPs in alleviating IBD are summarized in Figure 4.

EMMPs can effectively treat and prevent IBD by increasing the mucus layer and its secretion and promoting the expression of TJs such as junctional adhesion molecules, cytosolic scaffold proteins, and ZO-1, thereby improving the integrity of the colonic mucosal barrier. For example, in DSS-induced colitis mice, the polysaccharides from *I. obliquus* and Huangshan floral mushroom upregulated the TJ proteins occludin and ZO-1 and increased Muc-2 expression in colon tissue.^183,184^ Besides, both *T. fuciformis* and *Hericium caput-medusae* polysaccharides improved the symptoms of weight loss and ameliorated diarrhea, supplemented with increased mucus layer thickness and up-regulated Muc-2 and TJ expressions to restore the intestinal epithelial barrier integrity.^185,186^ By constructing the damaged intestinal mucosal barrier, dangerous molecules (endotoxins such as lipopolysaccharides) and harmful microorganisms can be prevented from passing through the mucosa, further protecting other intestinal barriers.^224^

EMMPs can regulate the intestinal immune system, alleviate oxidative damage, and exert anti-inflammatory effects in IBD. EMMPs regulate the expression of inflammatory cytokines through multiple signaling pathways, involving innate immunity (macrophage populations, natural killer, neutrophils, and DCs) and adaptive immunity (T and B cells).^225^ (1) EMMPs inhibit TLR4–NF-κB/MAPK and MANF-BATF2 signaling pathways to alleviate IBD. Lipopolysaccharides (LPS) can enter the blood through the damaged intestinal barrier, activate the TLR4 downstream NF-κB/MAPK signaling pathway, and promote the secretion of proinflammatory factors. *P. eryngii* polysaccharide strengthened the phosphorylation level of NF-κB and MAPK to enhance the immune regulation in LPS-treated RAW 264.7 macrophages.^48^ In LPS-induced intestinal inflammation mice, *F. velutipes* polysaccharide significantly depressed the release and mRNA expression of IL-1β, IL-6, IL-18, and TNF-α.^187^ Both the *H. erinaceus* polysaccharides with Mw 9.9 kDa and 86.67 kDa suppressed the activation of the NF-κB signaling pathway and decreased the colonic production of proinflammatory cytokines in DSS-induced colitis mice.^13,188^ Similarly, *F. velutipes* polysaccharide inhibited the TLR4/NF-κB pathway, decreased the pro-inflammatory cytokines, and increased the anti-inflammatory cytokines.^189^ As a secretion protein induced by endoplasmic reticulum stress, MANF can negatively regulate macrophage inflammation and BATF2, and BATF2 can upregulate the expression of NF-κB.^226^ Recently, it was found that the polysaccharide from *Boletus aereus* attenuated colitis and regulated the mucus barrier by increasing MANF expression and reducing BATF2 expression, targeting the MANF–BATF2–NF-κB signaling pathway.^190^ (2) EMMPs alleviate IBD by regulating helper T cell (Th) homeostasis. Th cells, including Th1, Th2, and Th17, and regulatory T cells (Treg) play a key roles in the immune process. Th17 cells can stimulate the intestine to secrete TNF-α, IL-1β, IL-17, IFN-γ, and other cytokines to aggravate the inflammatory process, while Treg cells play a key anti-inflammatory and immunomodulatory role.^227^ It was found that the polysaccharides from *I. obliquus* and Huangshan floral mushroom could promote Th1/Th2 and Th17/Treg homeostasis in DSS-induced colitis mice.^183,184^ Similarly, the polysaccharides from *Pycnoporus sanguineus* and *C. sinensis* restored intestinal mucosal immunity by regulating the differentiation and proportion of Th1, Th2, Th17, and Treg cells.^191,192^ (3) EMMPs improve IBD by regulating macrophage polarization. M1 macrophages can release numerous proinflammatory cytokines and promote Th1 and Th17 cell-regulated immune responses, leading to inflammatory damage to the intestinal tissues of IBD patients; while M2 macrophages can release immunosuppression factors (such as IL-10), activate Th2 immune responses, and inhibit Th1 immune responses, exerting anti-inflammatory effects.^228^ Research on the *D. indusiata* polysaccharide showed that it suppressed M1 macrophage polarization and promoted M2 macrophage polarization in the spleen and colon of colitis mice.^193^ (4) EMMPs alleviate IBD by alleviating oxidative stress. Typically, inflammatory responses are accompanied by oxidative stress, which markedly promotes the production of oxygen free radicals. The oxygen free radicals and the antioxidases in the intestine mainly include ROS, MPO, NO, MDA, plasma diamine oxidase (DAO), and GSH.^229^*Agaricus blazei* Murrill and *Pleurotus tuber-regium* polysaccharides significantly suppressed inflammation and oxidative stress.^194,195^*H. erinaceus* polysaccharide reduced the MDA level and increased the levels of antioxidases SOD and CAT in the colon of UC mice.^196^ MPO is an inflammatory marker of neutrophil infiltration. *P. eryngii* polysaccharide could suppress the accumulation of immune cells, macrophages, and neutrophils to alleviate colitis.^197^ iNOS can catalyze the production of NO after being stimulated by oxidative stress. It was found that *Phellinus linteus* polysaccharide inhibited the oxidative stress-related enzymes iNOS and MPO and enhanced the anti-inflammatory factors in ulcerative colitis mice.^198^

EMMPs can effectively modulate the homeostasis of intestinal microbiota, increase the diversity of intestinal microbiota, facilitate the production of SCFAs, and strengthen the intestinal microbial barrier. For mice with DSS-induced ulcerative colitis, polysaccharide from *F. velutipes* increased the microbial community diversity and decreased the ratio of Firmicutes and Bacteroidetes.^199^*A. auricula-judae* (Bull.) polysaccharide decreased the harmful bacteria *Ruminococcus*, Deferribacteres, and Actinobacteria.^200^ Besides, *T. fuciformis* polysaccharide raised the amounts of beneficial bacteria *Lactobacillus*, *Odoribacter*, *Helicobacter*, Ruminococcaceae, and Marinifilaceae.^201^ SCFAs bind to receptors such as GPR41, GPR43, and GPR109A, activating downstream signaling pathways like NF-κB and MAPK, stimulating the production of immunosuppressive cytokines, subsequently enhancing the intestinal mucosal barrier and maintaining intestinal homeostasis.^230^ EMMPs can promote the growth of SCFA-producing bacteria and promote the production of SCFAs, thereby exerting an immunomodulatory effect and alleviating enteritis.^202,203^

### Effect of EMMPs on Colorectal Cancer

Colorectal cancer (CRC) is a malignant tumor originating from the epithelium of the colon mucosa, and it is one of the most common malignant tumors in the gastrointestinal tract.^231^ CRC comprises two types, colon cancer and rectal cancer, with colon cancer accounting for 70%.^232^ A family history of colorectal cancer, chronic intestinal inflammation, a diet abundant in red meat and fat while lacking fruits and vegetables, obesity, alcohol consumption, and smoking are all possible factors that increase the incidence of CRC.^233^ Numerous studies have indicated that EMMPs exert potential anticancer benefits (Table 3).

EMMPs have anticancer properties by inhibiting tumor cell proliferation and inducing apoptosis. *G. lucidum* polysaccharide was found to possess antitumor activity through inhibiting migration and inducing apoptosis in LoVo human colon cancer cells.^204^ Another study found that *G. lucidum* polysaccharide induced autophagy and apoptosis in CRC-related cells HT-29 and HCT116 via activating the MAPK/ERK signaling pathway and inhibited tumor growth and autophagic flux in xenograft cancer bearing mice.^205^ Two kinds of polysaccharides from *H. erinaceus* aroused apoptosis in human colorectal cancer cells through the caspase-9-dependent signaling pathway^206^ and suppressed cancer cell growth by arresting the cell cycle in the S-phase.^207^ Furthermore, lentinan, entirely composed of d-glucose, could suppress tumor growth both *in vitro* and *in vivo* mainly through inducing autophagy and endoplasmic reticulum stress^208^ and showed antiproliferative and antitumorigenic properties in human colon cancer cell line HT-29.^209^

EMMPs possessed anti-inflammatory and antitumor functions, regulating the balance of inflammatory and anti-inflammatory immune cells. Research revealed that *G. lucidum* polysaccharide induced a shift of colon M1 macrophages to M2 type and promoted anti-inflammatory cytokines, accordingly strengthening the inflammatory intestinal barrier in colorectal cancer mice.^210^ Polysaccharides from *G. frondosa* reduced the proinflammatory cytokines COX-2, IL-6, and TNF-α and relieved oxidative stress through decreasing MPO activity and NO content and increasing the expression of GSH and SOD, and the mechanism of alleviating cancer was associated with the modulation of the Wnt/β-catenin/GSK-3β signaling pathway.^211^*L. edodes* polysaccharide inhibited lymphangiogenesis and lymphatic metastasis via regulating the TLR4/JNK signaling pathway in CRC mice.^212^ Activation of the NLRP3 inflammasome is relevant to inflammation-induced cancer. It was found that the *I. obliquus* polysaccharide promoted the colonic expression of the NLRP3 inflammasome, IL-1β, and IL-18 to ameliorate colon cancer in mice.^213^ Moreover, the activation of Th1 immunity highly contributed to the antitumor effects of *Sanghuangporus vaninii* polysaccharide.^214^

EMMPs also alleviated colorectal cancer by modulating gut microbiota and their metabolites. Polysaccharide from *S. vaninii* prevented dysbiosis in the gut microbiota associated with the l-arginine biosynthetic pathway.^214^*G. lucidum* polysaccharide improved intestinal dysbiosis by significantly reducing the relative abundance of *Oscillospira* and a genus of Desulfovibrionaceae in CRC mice.^215^ Besides, another study found that the *G. lucidum* polysaccharide ameliorated microbiota dysbiosis, promoted SCFA production, and alleviated endotoxemia to inhibit tumorigenesis in the colon.^216^

### Effect of EMMPs on Enteropathogenic Bacteria

Some pathogenic microorganisms caused bacterial infections in the intestines, significantly affecting intestinal health.^234^ EMMPs could inhibit the growth of many pathogens, further indicating their potential to resist intestinal infections caused by certain pathogens (Table 3). Heteropolysaccharides from *Ganoderma formosanum* protected mice from infection by *Listeria monocytogenes* characterized by stimulating macrophages to initiate an inflammatory response.^217^ A *Ganoderma applanatum* exopolysaccharide expressed antibacterial effects against *Staphylococcus aureus*.^218^ Polysaccharides isolated from liquid mycelial cultures of *L. edodes* exhibited antibacterial effects against *Salmonella* infection in mice by activating Th1 immune responses, with an improvement in IL-1β, IL-2, IL-6, and IL-12 levels.^219^ Sulfated polysaccharides isolated from *P. eryngii* inhibited *S. aureus*, *Escherichia coli*, and *L. monocytogenes*.^220^ Similarly, intracellular polysaccharides isolated from *G. frondosa* showed antibacterial effect against *E. coli*, *S. aureus*, *Bacillus megaterium*, and *L. monocytogenes*.^221^ Crude polysaccharide from *A. auricula-judae* had significant antimicrobial activities against *E. coli* and *S. aureus*.^222^

## Future Perspectives and Challenges

EMMPs are bioactive prebiotics that can pass the gut almost intact and directly reach the large intestine for further fermentation by the intestinal microbiota, thereby affecting the host’s nutrient absorption, energy expenditure, and immunologic function. This review briefly introduced the structure, digestion, absorption, and utilization pathways of EMMPs in order to explore how to achieve higher oral bioavailability of EMMPs. Gastrointestinal health has been the subject of numerous research. An increasing number of studies have found that the gastric barrier and microorganisms present in the stomach also play a protective role in human health. Therefore, this review summarized the structures and composition of the gastric and intestinal barriers and various factors that cause GI damage, emphasizing the important role of GI physical, chemical, immunological, and biological barriers. Furthermore, due to active components such as β-d-glucan in the EMMPs, they exhibited favorable therapeutic effects on gastrointestinal-related diseases after being digested and absorbed in the body. For the first time, this review summarized the gastroprotective effects of EMMPs against gastric mucosa injury, gastritis, gastric ulcer, and gastric cancer. In addition, intestinal protective effects of EMMPs on intestinal homeostasis, IBD, colorectal cancer, and intestinal infection were also summarized. Mechanistically, EMMPs exert GI protective effects by enhancing the physical, chemical, immunological, and biological barriers of the stomach and intestine.

The physical and chemical properties of EMMPs, especially their stereoselectivity, molecular weight, and structural diversity, have a significant impact on their therapeutic effects. Nevertheless, much remains to be learned about the relationships among the molecular weight, monosaccharide contents, glycoside binding form, and higher-order structure and gastrointestinal microecological effects of EMMPs. Therefore, future research should focus more on the relationship between the structure and function of EMMPs to further elucidate their mechanisms of action at the cellular and molecular levels, thereby promoting their potential applications in different areas such as dietary nutritional supplements, functional foods, or pharmaceuticals. In addition, developing economical and sustainable standardized isolation, purification, and production technologies for EMMPs is also a focus of future research.

As prebiotics, EMMPs can regulate the gut microbiota, which has been confirmed by numerous studies. However, through the summary in this review, it is found that the role of EMMPs in protecting the gastric barrier, regulating gastric flora, and protecting against gastric-related diseases cannot be ignored. Therefore, in future studies, more attention should be paid to the importance of EMMPs in the treatment of gastric diseases. Additionally, it is crucial to study the bioavailability and pharmacokinetics of EMMPs in animal models or the human body.^235^ On this basis, the digestion, absorption, and metabolism patterns of EMMPs in the gastrointestinal tract should be explored, thereby helping to better understand their functional mechanisms for protecting the gastrointestinal tract.

EMMPs are easy to obtain, have few side effects, and are considered clinically safe and well-tolerated. In a phase I open-label trial on 26 healthy adults supplementing active hexose correlated compound extract from *L. edodes*, 6 of 26 healthy adults showed nonsevere, transient symptoms like diarrhea, bloating, headache, foot cramps, and fatigue.^236^ Oral administration of soluble β-glucan is safe in healthy older adults and appears to induce an increase in circulating β-cells.^237^ Therefore, when preparing EMMPs, their safety should be fully considered, which depends on the source of mushrooms, extraction methods, and purity of polysaccharides. In the present, EMMPs can serve as dietary supplements, nutrition enhancers, edible films (packaging materials), etc. For example, the polysaccharide products from *C. sinensis* have been served as dietary supplements made by the manufacturer Maple Life Sciences, and the polysaccharide products from *G. lucidum* serve as an immunomodulator by Herbal Island.^238^ Interestingly, the polysaccharide from Polish wild mushroom promoted the growth of *Lactobacillus* strains stronger than commercially available prebiotics like inulin or fructooligosaccharides, also indicating its prebiotic potential.^239^ Besides, EMMPs have certain medicinal advantages over traditional drug treatments in preventing and treating gastrointestinal diseases. For example, *P. ostreatus* and *Trametes versicolor* have been made as medicinal products to improve digestion and provide relief from IBD.^238^ However, whether they can replace traditional treatments or achieve the same therapeutic effects still needs further exploration. Although an increasing number of *in vitro* and *in vivo* studies have determined the benefits of EMMPs, more large-scale, multicenter randomized controlled trials are needed in the future to further determine the safe and effective doses of EMMPs. More interestingly, precision nutrition and personalized medicine are also future research directions. Due to their excellent natural advantages such as hydrophilicity, tunability, and mechanical stability,^240−242^ EMMPs have been used as carriers for drug delivery. Biyang floral mushroom polysaccharides, as a novel type of nanoscale particle carrier in delivering resveratrol, exhibited good physicochemical stability, sustained gastrointestinal digestion release characteristics, and improved *in vitro* antioxidant and anticancer activities.^243^ Nanoparticles based on *G. lucidum* polysaccharides, as carriers for delivering bioactive compounds, targeted various cancer tissues and reduced chemotherapy-related side effects.^244^ Therefore, customizing personalized EMMPs or using EMMPs as a carrier to achieve precise nutrition based on the physical characteristics and gastrointestinal functional characteristics of different groups of people is also a new opportunity to promote the development of the big health industry.
